# Ternary Schottky Junction for Sonocatalytic Water Splitting in Gas‐Immunotherapy‐Mediated Cancer Treatment

**DOI:** 10.1002/advs.202413519

**Published:** 2025-02-08

**Authors:** Rui Zhang, Qian Wang, Junjie Pan, Jun Du, Han Yang, Bingfeng Wang, Yuhao Li, Yuqing Miao, Xumin Hou, Jingxiang Wu, Qing Miao

**Affiliations:** ^1^ Department of Anesthesiology Shanghai Chest Hospital School of Medicine Shanghai Jiao Tong University Shanghai 200030 China; ^2^ Institute of Bismuth Science School of Materials and Chemistry Shanghai Collaborative Innovation Center of Energy Therapy for Tumors University of Shanghai for Science and Technology Shanghai 200093 China; ^3^ College of Materials and Energy South China Agricultural University Guangzhou 510631 China

**Keywords:** gas therapy, glutathione consumption, immune activation, Schottky junction, water splitting

## Abstract

Hydrogen therapy has shown new potential in cancer treatment, particularly in high‐pressure and hypoxic areas, where it demonstrates the ability to alter the tumor microenvironment and regulate tumor metabolism. Hydrogen disrupts the mitochondrial function of cancer cells, interferes with their energy metabolism, and ultimately leads to energy depletion and apoptosis. In this study, a sonocatalyst (BPM), is designed to generate hydrogen and oxygen in situ within tumors, further enhancing the therapeutic efficacy. The mesocrystalline structure of BPM, composed of bismuth fluoride, polyoxometalates, and molybdenum carbide, significantly improves charge separation and electron transfer efficiency under ultrasound irradiation, resulting in an efficient water‐splitting reaction. By simultaneously generating hydrogen and oxygen within the tumor microenvironment and depleting glutathione, BPM effectively triggers oxidative stress and alleviates hypoxia, thereby disrupting mitochondrial function and inhibiting energy metabolism in cancer cells. Additionally, BPM enhances antitumor immune responses by promoting dendritic cell maturation, activating T lymphocytes, and polarizing macrophages toward the M1 phenotype, reversing the immunosuppressive state of the tumor microenvironment. The results indicate that BPM holds potential for gas‐immunotherapy combination treatments, offering a multifunctional strategy to improve cancer therapy outcomes.

## Graphical abstract

A sonocatalyst, BPM (Mo_2_C‐POM‐BiF_3_), is designed for in situ water splitting within tumors to generate H_2_ and O_2_, disrupting mitochondrial function, depleting GSH, and alleviating hypoxia. This enhances tumor cell apoptosis, inhibits proliferation, and activates antitumor immune responses by promoting dendritic cell maturation, T lymphocyte activation, and M1 macrophage polarization, offering a promising cancer therapy strategy.

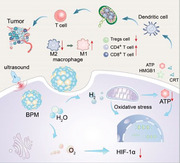

## 1 Introduction

Cancer treatment has long been a significant challenge in the global medical field.^[^ ^1^ ^]^ Although traditional therapies, such as radiotherapy and chemotherapy, can effectively inhibit tumor growth to a certain extent, they are often accompanied by severe side effects and can lead to tumor recurrence.^[^ ^2^ ^]^ In recent years, hydrogen (H_2_) therapy has attracted increasing attention as an adjunctive treatment due to its notable antioxidative, anti‐inflammatory, and cytoprotective effects.^[^ ^3^ ^]^ Hydrogen molecules can selectively neutralize harmful reactive oxygen species (ROS) and reactive nitrogen species, thereby reducing oxidative stress‐induced cellular damage and offering a mild and effective treatment option for cancer patients.^[^ ^3^, ^4^ ^]^ However, despite its potential advantages, the widespread application of hydrogen therapy in cancer treatment faces certain challenges, mainly due to its poor tissue permeability and rapid diffusion, which limit its ability to exert sustained effects in deep tissues.^[^ ^3^, ^4^, ^5^ ^]^

Researchers have developed various nanomaterials for hydrogen‐based cancer therapy, including H_2_‐carrying materials and in situ catalytic nanomaterials.^[^ ^3a,d^ ^]^ H_2_‐carrying materials release H_2_ slowly to prolong their action time in the body and enhance therapeutic effects. However, their H_2_‐carrying capacity is limited, and the bioavailability of these materials in tumors also restricts their development. Producing H_2_ in situ within the tumor via water splitting is an effective approach.^[^ ^3^, ^4^ ^]^ For instance, platinum‐based nanomaterials and titanium oxide have been shown to split water to generate H_2_ under light irradiation.^[^ ^3^, ^4^ ^]^ However, since light penetration depth is limited, ultrasound, which has a greater penetration depth, is an ideal choice for deep tumor treatment.^[^ ^3^, ^5^, ^6^ ^]^ Lin and Ma et al. proposed sonocatalytic water splitting to generate hydrogen for cancer treatment.^[^ ^3c^ ^]^ A Schottky heterojunction composed of platinum and bismuth sulfide was used for sonocatalytic water splitting, where H_2_ production was driven by water reduction, while the resulting holes oxidized and consumed glutathione (GSH), disrupting the redox balance in the tumor microenvironment (TME).^[^ ^7^ ^]^ Additionally, He et al. reported using ZnS nanoparticles to achieve sonocatalytic full water splitting into hydrogen and oxygen for immunoactivation in deep tumors.^[^ ^6^ ^]^ This suggests that water splitting to generate H_2_ and oxygen (O_2_) within the TME for cancer treatment is feasible.

Previous studies have shown that metal carbides, especially molybdenum carbide (Mo_2_C), possess semi‐metallic properties and can enhance semiconductor catalytic efficiency by acting as noble metals.^[^ ^8^ ^]^ Feng et al. encapsulated molybdenum carbide quantum dots in ultrathin nitrogen‐doped graphene vesicles, demonstrating superhigh H_2_ production from pure water.^[^ ^9^ ^]^ Forming a composite structure to regulate electron transfer and band structure was found to improve hydrogen evolution reaction (HER) performance.^[^ ^8a,b^ ^]^ Therefore, tuning the bandgap of Mo_2_C may enable effective water splitting. Polyoxometalates (POMs), known for their strong redox activity, can act as electron donors or acceptors when combined with semiconductor materials, enhancing photoelectrochemical performance.^[^ ^10^ ^]^ Bismuth‐based materials are semiconductors with tunable bandgaps that provide excellent electron–hole separation and reduce the recombination rate of electrons and holes.^[^ ^2^, ^11^ ^]^ Moreover, bismuth‐based materials have shown multiple activation properties for cancer catalytic treatment, serving as drug delivery vehicles or catalysts with high biocompatibility.^[^ ^12^ ^]^

**Scheme 1:**
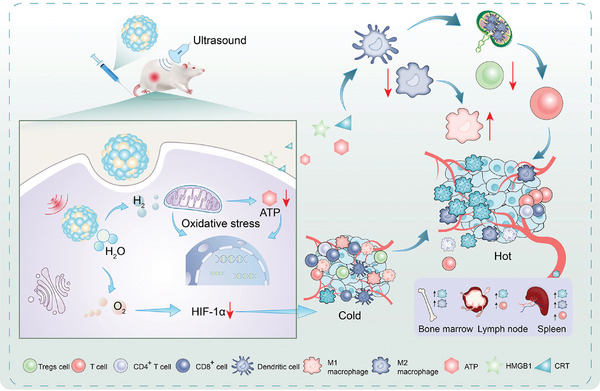
Schematic diagram of the mechanism of BPM used for sonocatalytic gas therapy to activate immune synergistic therapy.

Based on this, we designed a sonocatalyst for water splitting, composed of a ternary heterojunction with a mesocrystalline structure formed from Mo_2_C, POMs, and bismuth fluoride (Mo_2_C‐POM‐BiF_3_, or BPM) (**Scheme** **1**). In this catalyst, bismuth fluoride (BiF_3_), with its high bandgap and stability, facilitates effective charge separation; Mo_2_C, with its excellent conductivity and narrow bandgap, accelerates electron transfer and H_2_ evolution; and POMs provide multiple active sites, forming efficient charge separation and transfer pathways. Upon ultrasound (US) activation, sonogenerated electrons are transferred from the surface of BiF_3_ through POM to the Fermi level of Mo_2_C via the Schottky junction, significantly enhancing electron transfer efficiency and suppressing carrier recombination. BPM can split water in situ within tumors to generate H_2_ and O_2_, while BiF_3_ degrades by consuming GSH. BPM inhibits cellular energy metabolism, alleviates tumor hypoxia, and disrupts the redox balance in the TME, inducing tumor cell apoptosis and inhibiting proliferation. Furthermore, this multifunctional strategy synergistically suppresses tumor growth in vivo by increasing the infiltration of dendritic cells, T cells, and M1 macrophages in the TME, while reducing the percentage of M2 macrophages, thus triggering a strong antitumor immune response. This system offers an effective catalyst design strategy for in situ sonocatalytic water splitting in tumors for H_2_‐based immunotherapy activation.

## 2 Results and Discussion

### 2.1 Synthesis and Characterization of BPM

**Figure 1:**
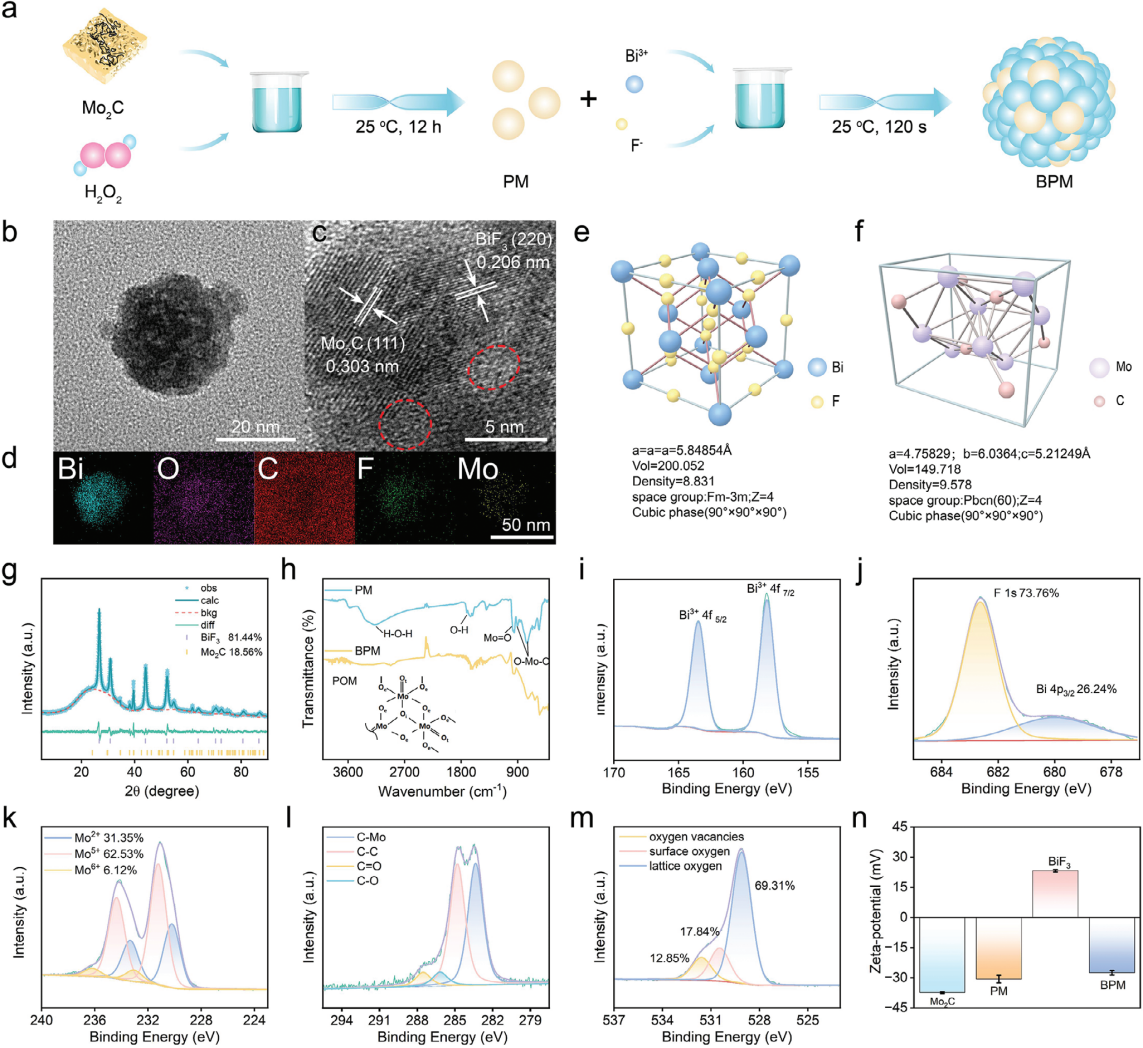
Synthesis and characterization of BPM. a) Schematic diagram of the synthesis process of BPM, b) TEM image, c) high‐resolution TEM image (marked in red circles as the amorphous phase region), d) element mapping image containing elements Bi, O, C, F, and Mo. The cell structures and parameters of e) BiF_3_ and f) Mo_2_C in BPM were refined by the Rietveld method. g) X‐ray diffraction pattern of BPM and its fitting refinement results. h) Fourier transform infrared spectra of BPM and PM. High‐resolution XPS spectra of i) Bi 4f, j) F 1s, k) Mo 3d, l) C 1s, and m) O 1s elements in BPM. n) Zeta potentials of Mo_2_C, PM, BiF_3_, and BPM.

A ternary heterojunction BiF_3_‐POM‐Mo_2_C (BPM) was synthesized via oxidation and precipitation methods (**Figure** **1**a; Figure S1, Supporting Information). Transmission electron microscopy (TEM) revealed that the diameter of POM‐Mo_2_C (PM) particles was below 10 nm, forming a near‐spherical structure (Figure S2, Supporting Information). The synthesized BPM nanoparticles were ≈40 nm in diameter (Figure 1b), consisting of small spheres with a mesocrystalline structure, which increases the ultrasonic absorption cross‐section and improves catalytic performance. The Brunauer−Emmett−Teller (BET) results showed a specific surface area of 19.262 m^2^ g^−1^ and an average pore size of 1.4 nm (Figure S3, Supporting Information). In Figure 1c, high‐resolution TEM images showed that the lattice spacing of BiF_3_ was 0.206 nm, corresponding to the (220) plane of the face‐centered cubic crystal phase of BiF_3_, and the lattice spacing of Mo_2_C was 0.303 nm, corresponding to the (111) plane of the orthorhombic crystal phase of Mo_2_C. Red markers indicate some amorphous phases, likely due to the embedding of amorphous POM within the material. Energy dispersive X‐ray spectroscopy element mapping confirmed the uniform distribution of Bi, O, C, F, and Mo elements throughout the nanocomposite material (Figure 1d). X‐ray diffraction Rietveld refinement analysis confirmed the cell parameters and structure of BiF_3_ and Mo_2_C (Figure 1e–g). The diffraction peaks of the (111), (200), (220), and (311) planes matched the cubic phase of BiF_3_ (CDS No.24522), and the diffraction peaks of the (110), (020), (121), and (102) planes matched the orthorhombic phase of Mo_2_C (CDS No.164851), consistent with the lattice spacings measured in high‐resolution TEM.

Fourier‐transform infrared spectroscopy (Figure 1h) and X‐ray photoelectron spectroscopy (XPS) were used to analyze the elemental composition of BPM and deduce the structure of POM. In the high‐resolution Bi XPS spectrum, the binding energy peaks at 163.5 and 158.1 eV were attributed to Bi^3+^ 4f_5/2_ and Bi^3+^ 4f_7/2_, respectively (Figure 1i). The binding energy of F^−^ in the 2p orbital was 682.6 eV (Figure 1j). The Mo 3d orbital signals could be fitted to six characteristic peaks corresponding to three valence states of Mo ions: Mo^2+^, Mo^5+^, and Mo^6+^, with ratios of 31.35%, 62.53%, and 6.12%, respectively. Mo^2+^ originated from Mo_2_C, while Mo^5+^ and Mo^6+^ were mainly derived from POM, with Mo^5+^ dominating (Figure 1k). The binding energy peak at 283.3 eV in the high‐resolution C XPS spectrum matched the characteristic peak of C–Mo, confirming the presence of Mo_2_C (Figure 1l). The two main peaks at 531.5 and 530.4 eV in the high‐resolution O XPS spectrum were attributed to oxygen vacancies and surface‐adsorbed oxygen, which facilitate carrier migration and inhibit carrier recombination (Figure 1m). Dynamic light scattering measurements showed that the size of BPM was 120 nm (Figure S4, Supporting Information). Before composite formation, the zeta potentials of PM and BiF_3_ were −30.6 and 23.3 mV, respectively. After composite formation, the zeta potential of the mixture became −27.3 mV (Figure 1n), further confirming the construction of the BPM ternary heterojunction. BPM could be stored in slight acid phosphate buffer saline (PBS) for several days, conforming to its stability (Figure S5, Supporting Information).

### 2.2 Water Splitting of BPM by US and GSH Consumption

**Figure 2:**
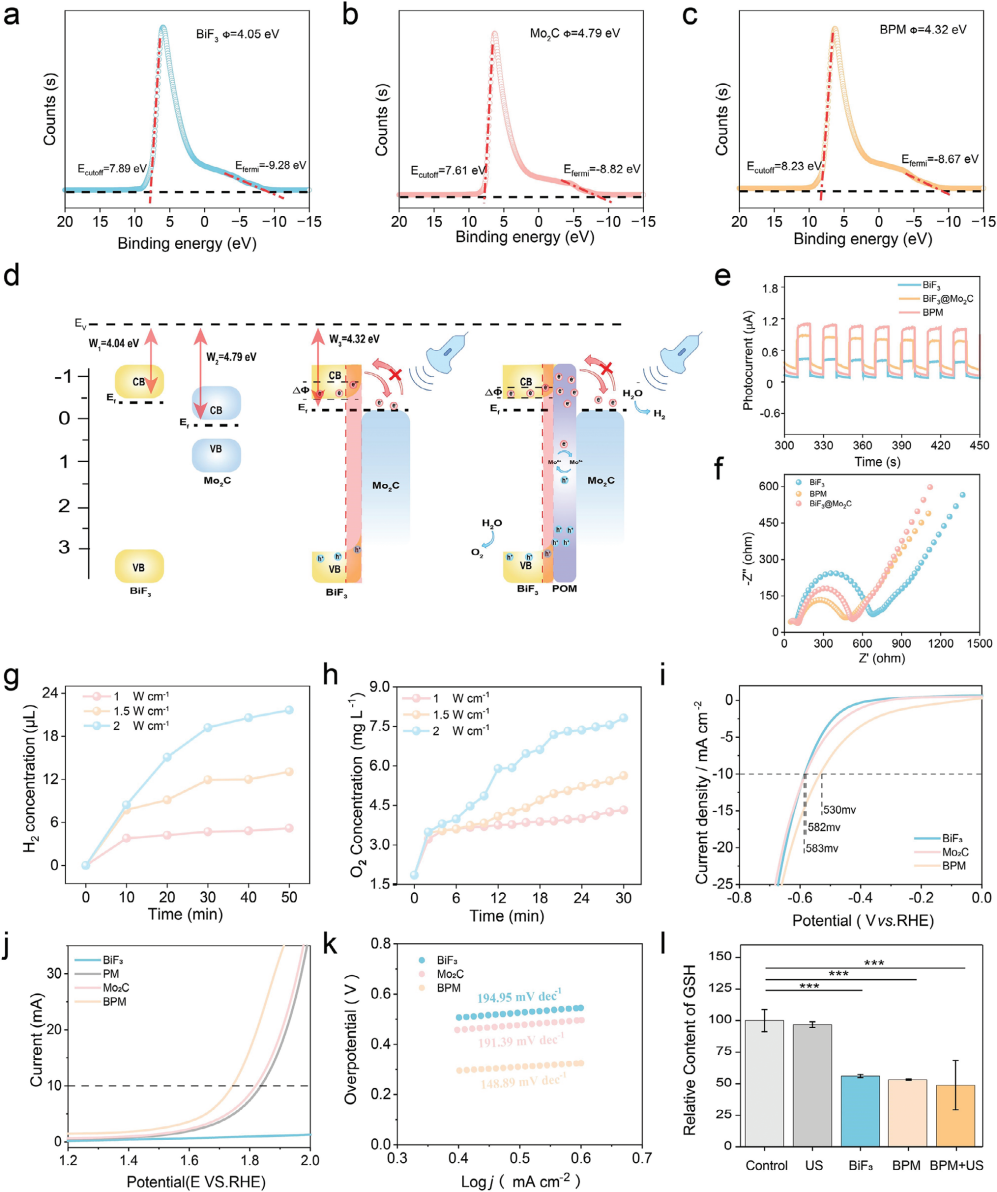
Water splitting performance and mechanism evaluation of BPM. Ultraviolet photoelectron spectra of a) BiF_3_, b) Mo_2_C, and c) BPM. d) Band diagrams and sonocatalytic mechanisms of BiF_3_, Mo_2_C, BiF_3_‐Mo_2_C, and BPM. e) Time‐dependent photocurrent response and f) electrochemical impedance spectroscopy of BiF_3_, BiF_3_‐Mo_2_C, and BPM. g) The hydrogen evolution curve and h) the oxygen production curve of BPM with time under different US power excitation. Linear sweep voltammetry i) HER and j) OER of BiF_3_, Mo_2_C, and BPM. k) Tafel plots of BiF_3_, Mo_2_C, and BPM from HER linear sweep voltammetry curves. l) GSH consumption in different treatment groups. Student's t‐test, *p < 0.05, **p < 0.01, and ***p < 0.001. Data are presented as mean ± SD (*n* = 5).

The semiconductor mechanism of BPM sonocatalytic water splitting for H_2_ and O_2_ generation was investigated. Ultraviolet photoelectron spectroscopy was used to measure the work function (W) and Fermi level (E_f_) of BiF_3_, Mo_2_C, and BPM to determine the direction of charge carrier transfer in the heterojunction. The difference in E_f_ between the semiconductors is the intrinsic driving force for charge transfer in the heterostructure. As shown in **Figure** **2**a–c, the work functions of BiF_3_, Mo_2_C, and BPM were calculated to be 4.05, 4.79, and 4.32 eV, respectively, while their Fermi levels were −0.45, 0.29, and −0.18 eV, respectively. Mo_2_C has a larger W and lower E_f_, whereas BiF_3_ has a smaller W and higher E_f_. When BiF_3_ is in close contact with Mo_2_C, electrons transfer from BiF_3_ to Mo_2_C. Since previous studies have indicated that Mo_2_C exhibits pseudo‐noble metal properties, a space charge region forms on the BiF_3_ side when BiF_3_ and Mo_2_C are in close contact, causing band bending and forming a Schottky barrier. When excited by US, the electrons in BiF_3_ are excited from the valence band (VB) to the conduction band (CB) and transfer through the Schottky barrier to the Fermi level of the Mo_2_C nanoparticles, while the holes remain in the VB of BiF_3_ (Figure 2d). Importantly, the band bending effectively prevents electron backflow, further accelerating carrier separation in the BPM catalytic system.

Electrochemical impedance and transient photocurrent measurements were used to verify carrier separation efficiency and migration capacity. As shown in Figure 2e,f, electrochemical impedance spectroscopy results revealed that the Nyquist semicircles represent the electron transfer resistance in the materials. BPM exhibited smaller Nyquist semicircles and higher photocurrent density compared to BiF_3_‐Mo_2_C and BiF_3_, indicating that the formation of the Schottky junction successfully reduced charge transfer resistance. More electrons and holes could reach the reaction interface upon irradiation, generating higher transient photocurrent density. Notably, BPM's impedance and photocurrent data were superior to those of BiF_3_‐Mo_2_C, likely due to the ability of POM to rapidly switch between multiple valence states, enhancing charge transfer efficiency when POM acts as an electron transfer medium. Furthermore, we utilized ultrasound to excite BPM, BiF_3_, and BiF_3_‐Mo_2_C, BPM also presented the most obvious sonocurrent response, verifying the ability of sono‐excited charge separation (Figure S6, Supporting Information).

To determine whether the sonogenerated electrons and holes had sufficiently high redox potential for HER and oxygen evolution reaction (OER), the band structures of the nanomaterials were further measured using Mott–Schottky and solid‐state diffuse reflectance spectroscopy (Figure S7, Supporting Information). The bandgaps (Eg) of BiF_3_ and Mo_2_C were 3.52 eV and 0.46 eV, respectively. The calculated band structures of BiF_3_ and Mo_2_C (Figure S8, Supporting Information) exhibited typical direct bandgap semiconductor and metallic characteristics, respectively. The theoretical bandgap of BiF_3_ (3.81 eV) was slightly larger than the experimental value (3.52 eV). The theoretical bandgap of Mo_2_C was 0 eV, with the density of states of Mo d‐orbitals extending across the valence band and conduction band, allowing d‐electrons to move freely between the bands. By drawing tangent lines from the Mott–Schottky plots, the flat band potentials (E_fb_) of BiF_3_ and Mo_2_C were determined to be −0.98 and −0.5 eV (vs Ag/AgCl, pH 7), respectively. Therefore, the CB potentials of BiF_3_ and Mo_2_C were −0.48 and 0 eV (vs NHE, pH = 0), respectively. The CB potentials of BiF_3_ and Mo_2_C are more negative than the redox potential of H^+^/H_2_ (0 V vs NHE, pH 0). The VB potential of BiF_3_ is also more positive than the redox potential of H_2_O/O_2_ (1.23 V vs NHE, pH 0). Thus, BPM meets the conditions for water splitting to produce H_2_ and O_2_. Besides, after contact between H_2_O and BPM, the contact angle was only 27.4°, indicating that BPM has hydrophilicity and meets the conditions for water splitting (Figure S9, Supporting Information).

Subsequently, gas chromatography and a dissolved oxygen meter were used to obtain the H_2_ and O_2_ production of BPM. As shown in Figure 2g,h, and Figure S10 (Supporting Information), H_2_ was detected at different US power densities (1 MHz, 1, 1.5, and 2 W cm^−2^), with H_2_ production increasing with US power densities and excitation time. Simultaneously, O_2_ was detected. Regardless of US power densities, the molar ratio of H_2_ to O_2_ was ≈2:1, indicating that full water splitting occurred under US. Since the tumor‐sensitive concentration is in the nanomolar range, the gases produced by BPM are sufficient to exert therapeutic effects in tumors. Linear sweep voltammetry was used to measure the overpotentials for HER and OER (Figure 2i,j). BPM exhibited lower overpotentials than BiF_3_ and Mo_2_C. At a current density of 10 mA cm^−2^, the overpotentials of BPM, BiF_3_, and Mo_2_C were 530, 582, and 583 mV, respectively, indicating that BPM had the highest H_2_ production efficiency. Additionally, BPM exhibited a lower Tafel slope of 148.9 mV dec^−1^, further demonstrating its superior catalytic efficiency (Figure 2k). At a current density of 10 mA cm^−2^, BPM also had the lowest OER overpotential of 1.74 V, lower than that of Mo_2_C (1.82 V). Therefore, BPM enables simultaneous HER and OER under sonocatalysis, achieving water splitting for gas therapy.

Furthermore, the interaction between BPM and GSH was evaluated using 5,5′‐dithiobis‐(2‐nitrobenzoic acid) (DTNB) probes. As shown in Figure 2l, US alone did not affect GSH, while BiF_3_ and BPM effectively consumed GSH after 30 min of incubation, indicating that the GSH consumption was due to the presence of BiF_3_ in BPM. Under US activation, BPM did not further consume GSH, suggesting that all the holes generated by BPM under sonocatalysis were used for OER, with no significant effect on GSH. Therefore, GSH consumption disrupts the redox balance in the TME, exacerbating oxidative damage to the tumor. Besides, under TME conditions (pH 5.5, H_2_O_2_, and GSH), BPM could degreed over time, presenting a biodegradable characteristic (Figure S11, Supporting Information).

### 2.3 In Vitro Therapeutic Evaluation

**Figure 3:**
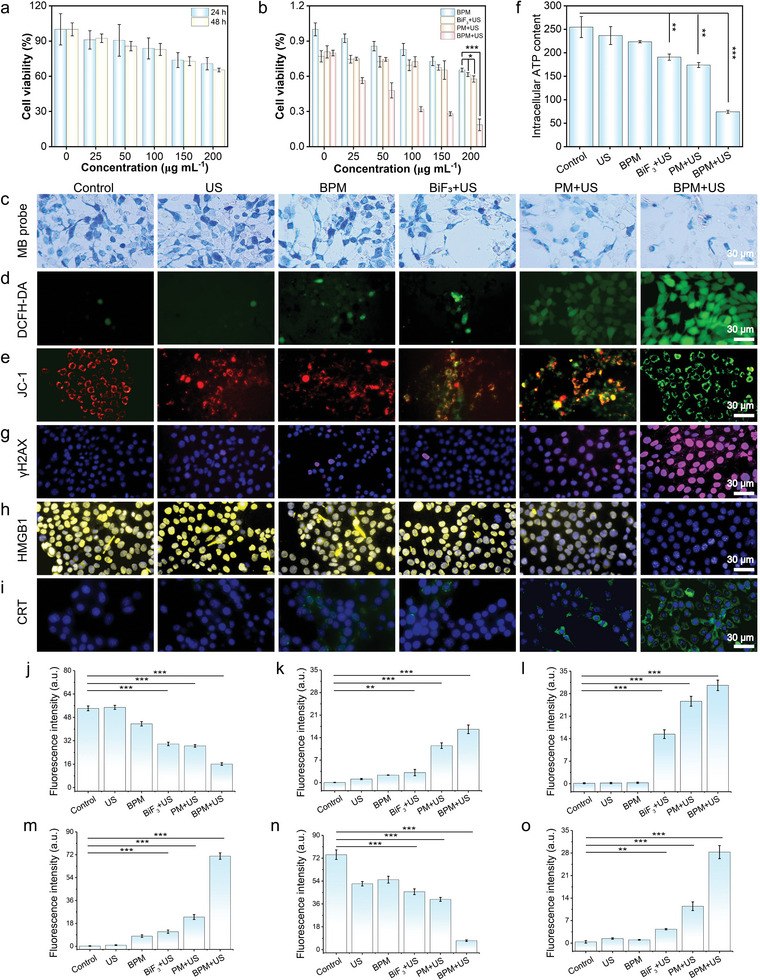
Evaluation of the in vitro tumor treatment effect of BPM. a) Cell viability of 4T1 cells after co‐incubation with BPM for 24 and 48 h, respectively. b) The cytotoxicity of 4T1 cells in different treatment groups. c) Images of 4T1 cells stained with MB probe after treatment in different groups. Fluorescence images of 4T1 cells stained with d) DCFH‐DA and e) JC‐1 after treatment in different groups. f) The ATP content inside 4T1 cells in different groups after treatment. Immunofluorescence staining images of (g) DNA damage, h) HMGB1, and i) CRT after treatment in different groups. j) MB, k) DCFH‐DA, l) JC‐1, m) γ‐H2AX, n) HMGB1, o) CRT corresponding fluorescence quantitative data. Student's *t*‐test, *p < 0.05, **p < 0.01, and ***p < 0.001. Data are presented as mean ± SD (*n* = 5).

The biocompatibility of BPM was assessed using the cell counting kit‐8 (CCK‐8). Mouse breast cancer (4T1) and human embryonic kidney (293T) cells were incubated with different doses of BPM for 24 and 48 h. At concentrations below 100 µg mL^−1^, cell viability was not significantly inhibited, indicating good safety of BPM. When the concentration is higher than 100 µg mL^−1^, cell viability in 4T1 is lower than that in 293T cells, which could be caused by GSH consumption and the catalytic performance of Mo ions (**Figure** **3**a; Figure S12, Supporting Information). As shown in Figure 3b, irradiation of 4T1 cells with US alone (0.7 W cm^−2^, 5 min) had minimal impact on cell viability. However, when 4T1 cells were incubated with BPM and then exposed to US, cell viability was significantly reduced, and this reduction was positively correlated with the concentration of BPM. This is due to oxidative damage caused by H_2_ and GSH consumption, which, combined with oxygen's ability to alleviate tumor hypoxia, synergistically induced apoptosis in cancer cells. Additionally, we evaluated the viability of BiF_3_ and PM under US, and their effects were weaker than that of BPM.

To further investigate the mechanism of BPM‐induced cell death, a methylene blue (MB) probe was used to analyze intracellular H_2_ release. As shown in Figure 3c,j, after co‐incubation with MB probes, the cells displayed a blue color. Compared to the control group, the blue color in cells treated with US, BPM alone, or BiF_3_+US remained largely unchanged. However, in the BPM+US treatment group, a significant fading of the blue color was observed in 4T1 cells, indicating that BPM effectively produced H_2_ within cancer cells. GSH consumption disrupts the oxidative balance within tumor cells, leading to oxidative stress in the tumor. Using 2′,7′‐dichlorodihydrofluorescein diacetate (DCFH‐DA) as a probe to monitor changes in intracellular ROS levels, significant ROS production was observed in cells treated with BPM+US (Figure 3d,k). Previous studies have shown that H_2_ can effectively target mitochondria within cells.^[^ ^3^, ^6^ ^]^ As such, H_2_ induces apoptosis in cancer cells by inhibiting mitochondrial function and blocking adenosine triphosphate (ATP) synthesis. Mitochondrial membrane potential is a core indicator of mitochondrial health, and apoptosis is often accompanied by a reduction in mitochondrial membrane potential. JC‐1, a mitochondrial membrane potential assay kit, was employed to measure changes in mitochondrial membrane potential. When mitochondrial membrane potential is high, JC‐1 forms red fluorescent aggregates in the mitochondrial matrix. Conversely, when mitochondrial membrane potential is low, JC‐1 exists as green fluorescent monomers. As shown in Figure 3e,l, the BPM+US group displayed significant green fluorescence, indicating that the generated H_2_ effectively damaged the mitochondria of 4T1 cells, which also led to a reduction in intracellular ATP levels (Figure 3f). Furthermore, reduced ATP levels and elevated ROS levels within cells may also cause DNA damage. By detecting the expression of γ‐H2AX protein, we investigated the mechanism of DNA double‐strand breaks. As shown in Figure 3g,m, the BPM+US treatment group exhibited the highest fluorescence intensity among all groups. These findings suggest that the produced H_2_ can induce mitochondrial and DNA damage, inhibit tumor cell proliferation, and lead to cell apoptosis.

Subsequently, we examined the expression of immunogenic cell death (ICD) biomarkers induced by gas therapy, including calreticulin (CRT) and high‐mobility group box 1 (HMGB1). These biomarkers can stimulate dendritic cell maturation and activate cytotoxic T lymphocytes, thereby enhancing antitumor immune responses. The expression of CRT and HMGB1 in 4T1 cells was visualized using CRT/HMGB1‐specific antibodies for immunofluorescence staining (Figure 3h,i,m,n). It was found that the HMGB1 signal was significantly reduced in the BPM+US group compared to other experimental groups, while the CRT signal displayed a negative correlation with HMGB1 expression. These data suggest that the gas therapy combination of oxidative damage caused by H_2_ and GSH consumption, along with O_2_ alleviating hypoxia, can trigger a certain level of ICD, thereby eliciting an immune response against tumor cells. Thus, in tumor cells, BPM can inhibit cell viability, regulate the TME through HER and OER, damage tumor cells, and induce ICD under US activation.

### 2.4 In Vivo Therapeutic Evaluation

Before evaluating the in vivo therapeutic effects, the systemic toxicity of BPM was assessed. Even at a high concentration of 200 µg mL^−1^, the hemolysis rate of BPM was below 5%, indicating that the BPM composite nanomaterials have satisfactory blood compatibility (Figure S13, Supporting Information). Healthy female BALB/c mice were intravenously injected with BPM (2 mg mL^−1^, 100 µL). Fourteen days after administration, there were no significant differences in the complete blood count data of the BMP group compared to the control group (Figure S14, Supporting Information). Meanwhile, the histological hematoxylin and eosin (H&E) staining of the major organs (heart, liver, spleen, lungs, kidneys) of mice sacrificed on day 14 also showed no detectable signs of damage or inflammatory lesions, indicating that BPM has high tissue compatibility.

**Figure 4:**
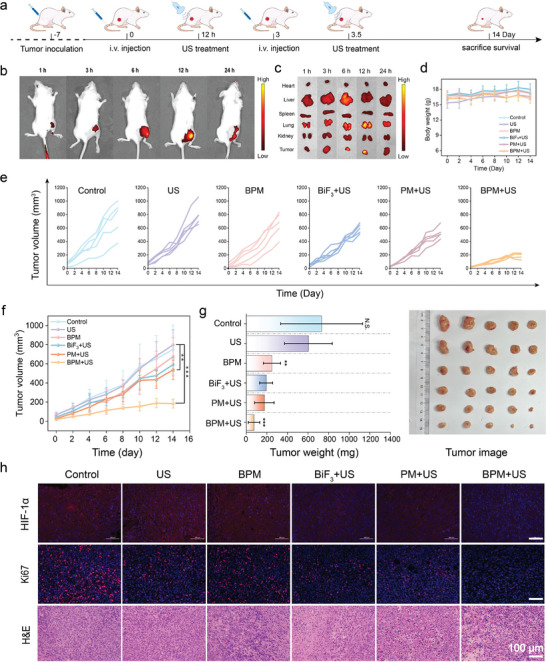
In vivo treatment evaluation of BPM. a) Schematic diagram of tumor establishment and treatment plan. After intravenous injection of BPM‐IR780, 4T1 tumor‐bearing mice showed fluorescence images of b) in vivo and c) in various organs and tumors over time. d) The weight of mice in different groups during treatment. The e) growth curves and f) average growth curves of tumor‐bearing mice in each group during the treatment period. g) The average weight and photos of tumors in each group after treatment. h) The slice images of HIF‐1α staining, Ki‐67 staining, and H&E staining in each group of tumors. Student's *t*‐test, *p < 0.05, **p < 0.01, and ***p < 0.001. Data are presented as mean ± SD (*n* = 5).

The treatment process is illustrated in **Figure** **4**a. To evaluate the uptake of BPM by the tumor and its biodistribution in various organs, fluorescence imaging was used (Figure 4b,c). IR780 (a near‐infrared dye) was loaded into BPM as a fluorescent indicator to obtain BPM‐IR780, which was injected into tumor‐bearing 4T1 mice via the tail vein. Twelve hours after injection, the relative distribution of BPM in the tumor reached its maximum, indicating that BPM effectively accumulated and retained in the tumor through circulation. Thus, 12 hours post‐injection was determined to be the optimal time for US treatment. The fluorescence intensity in the liver and kidneys first increased and then decreased over time, indicating that BPM could be metabolized.

Based on the therapeutic effects observed in cells, we further investigated the antitumor activity of BPM in a 4T1 tumor‐bearing mouse model. A total of 32 female BALB/c mice were randomly divided into six groups (*n* = 5): (1) Control, (2) US, (3) BPM, (4) BiF_3_+US, (5) PM+US, and (6) BPM+US. Treatment conditions: BPM, BiF_3_, and PM (2 mg mL^−1^, 100 µL) were intravenously injected into groups (3)–(6); US (0.7 W cm^−2^, 5 min) was applied. As shown in Figure 4a, treatment began 12 h after material injection on days 0 and 3, with a total of two treatments over 14 days. During the 14‐day treatment, there were no significant differences in the body weights of mice across all groups, indicating that the treatment did not affect body weight and had no obvious side effects (Figure 4d). The tumor volume growth curves (Figure 4e,f) showed that the tumors in the Control and US groups grew rapidly without being affected. BPM alone exhibited a certain inhibitory effect on tumor proliferation. The BiF_3_+US and PM+US groups also showed some inhibitory effects. In comparison, the BPM+US group exhibited the most significant tumor suppression. At the end of the treatment, the tumors were photographed and weighed (Figure 4g). Compared to the control group, the tumor masses in the BiF_3_+US, PM+US, and BPM+US groups were significantly lower, with the BPM+US group showing the lowest tumor mass. Notably, in some mice in the BPM+US group, the tumors had gradually regressed, further demonstrating the tumor‐suppressive effect of gas combination therapy. After treatment, H&E and Ki67 staining of tumor tissues showed that the BPM+US group had the highest degree of tumor cell death, the most significant morphological changes, and the greatest inhibition of proliferation. Hypoxia inducible factor 1α (HIF‐1α) staining further confirmed that O_2_ produced by BPM through the OER effectively relieved hypoxia within the tumor, reducing resistance to tumor immunotherapy (Figure 4h). Therefore, BPM exhibited superior tumor treatment efficacy compared to BiF_3_ and PM.

### 2.5 Evaluation of BPM‐Induced Immune Response in Tumor Cells

**Figure 5:**
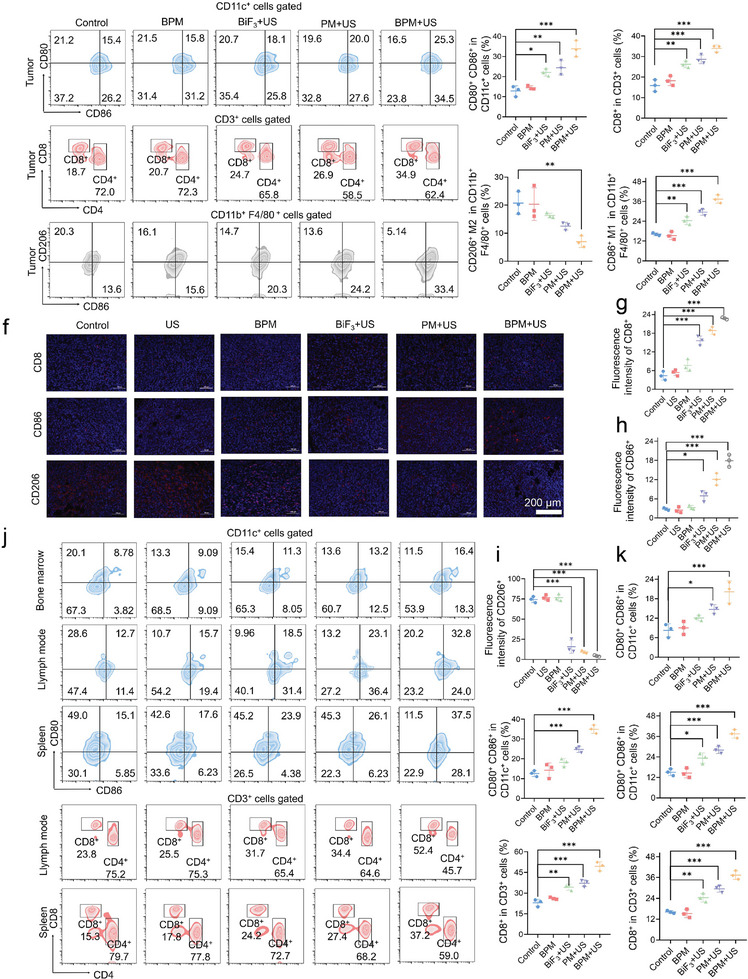
a) Flow cytometry result of mature DC cells, T cells, and macrophages in tumors, and corresponding analysis diagrams of b) mature DC cells, c) T cells, d) M2 type macrophages, and e) M1 type macrophages. f) Results of immunofluorescence staining of tumor sections, and corresponding average fluorescence intensity analysis diagrams of g) CD8 T cells, h) CD86 DC cells, and i) CD206 macrophages. (j) Flow cytometry result of mature DC cells in bone, lymph, and spleen, and corresponding analysis diagrams of mature DC cells in k) bone, l) lymph, and m) spleen. n) Flow cytometry result of T cells in lymph, and spleen, and corresponding analysis diagrams of T cells in o) lymph, and p) spleen. Student *t*‐test, *p < 0.05, **p < 0.01, ***p < 0.001. Data are presented as mean ± SD (*n* = 5).

Since BPM demonstrated the ability to induce ICD by triggering the release of various damage‐associated molecular patterns in cell therapy, we evaluated BPM's ability to activate immune responses after in vivo treatment. The proportion of mature dendritic cells (DCs) and activated T lymphocytes in the treated tumor tissues was quantified by flow cytometry. The gating strategy for flow cytometry is shown in Figures S15–S17 (Supporting Information). As there were no significant differences in therapeutic effects between the Control and US groups in the in vivo and in vitro experiments, we only evaluated (1) Control, (2) BPM, (3) BiF_3_+US, (4) PM+US, and (5) BPM+US groups. The co‐expression of CD80^+^ and CD86^+^ was used as a marker for mature DCs. In the BPM+US group, the proportion of mature DCs was significantly higher than in the control group, with an increase of 9.9% (**Figure** **5**a,b). At the same time, compared to the control group, the proportion of CD8^+^ T cells, which directly kill tumor cells, increased by 16.2% (Figure 5a,c). Additionally, numerous studies have shown that tumor hypoxia is considered one of the most important factors in immune suppression.^[^ ^6^, ^13^ ^]^ Hypoxic regions within solid tumors are infiltrated by a large number of immunosuppressive cells, such as M2‐type tumor‐associated macrophages, which suppress immune responses.^[^ ^3^, ^6^, ^14^ ^]^ Here, we found that compared to the control group, BPM+US treatment reduced the proportion of anti‐inflammatory CD206^+^ M2 macrophages and increased the proportion of pro‐inflammatory CD86^+^ M1 macrophages (Figure 5a,d,e). This demonstrated that BPM, activated by US, effectively relieved hypoxia in tumor sites and reversed immune suppression. The same conclusions were also supported by immunofluorescence images of CD86^+^, CD8^+^, and CD206^+^ in tumor tissues from the treatment groups (Figure 5f–i).

When ICD occurs in tumor cells, immature DCs recognize the release of damage‐associated molecular patterns, leading to DC maturation, cytotoxic T lymphocyte activation, and the subsequent induction of systemic immune activation to fight tumor metastasis. To evaluate this immune response, we assessed the maturation of DCs in the bone marrow, spleen, and lymph nodes. The proportion of mature DCs in the bone marrow, spleen, and lymph nodes in the BPM+US group was 16.4%, 37.5%, and 32.8%, respectively, which were significantly higher than those in the other groups (Figure 5j–m). Additionally, we analyzed the activation of CD8^+^ T cells. As shown in Figure 5n–p, the activation rates of CD8^+^ T cells in the spleen and lymph nodes in the synergistic treatment group were 34.7% and 52.4%, respectively, showing significant enhancement compared to the control group. This indicates that the BPM+US group achieved systemic immune activation, leading to the most effective tumor suppression. These results suggest that under US stimulation, BPM effectively kills tumor cells by producing H_2_ and O_2_, consuming endogenous GSH, and significantly activating the immune system through the reversal of immune suppression, thereby enhancing the therapeutic efficacy of gas immunotherapy.

### 2.6 RNA‐Seq Analysis of BPM Synergistic Tumor Treatment Mechanism

**Figure 6:**
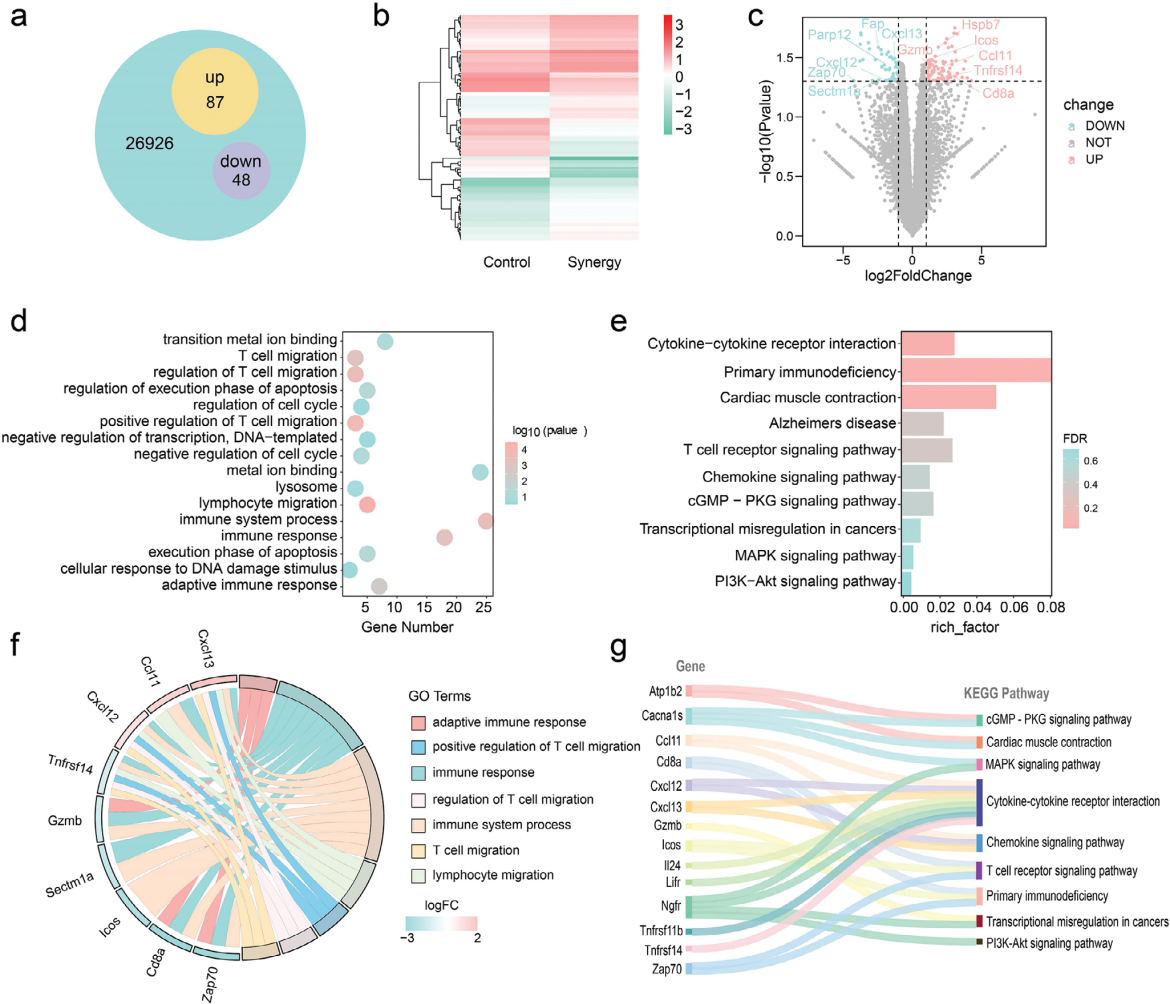
Investigates the therapeutic mechanism of BPM through transcriptome high‐throughput sequencing. a) Venn plot of DEGs in RNA seq, b) DEGs volcano plot, and c) DEGs heatmap between the control group and BPM+US (Synergy) treatment group. d) Significantly enriched GO terms. e) KEGG pathway enrichment analysis of DEGs. f) Analyze the correlation between the GO enrichment pathway and DEGs. g) Analyze the correlation between the KEGG enrichment pathway and DEGs.

To further explore the mechanism behind the gas‐immunotherapy killing effect of BPM under US stimulation, we analyzed mRNA expression in tumor tissues from the Control group and the BPM+US group using high‐throughput sequencing. A Venn diagram showed that 26 926 co‐expressed genes were found in both the control and BPM+US groups (**Figure** **6**a). Compared to the control group, ≈105 genes were abnormally expressed in 4T1 cells in the synergistic group, including 87 upregulated genes and 48 downregulated genes. A genomic heatmap illustrated the results of the differentially expressed genes (DEGs) (Figure 6b). The DEGs were identified using screening criteria (Log2FoldChange ≥ 1.0 or ≤ 1.0, *p*‐value ≤ 0.05) (Figure 6c). BPM+US treatment induced the downregulation of the proliferation‐promoting gene SECTM1, the DNA repair‐promoting gene Fap, and the tumor growth‐promoting factor Parp12, while upregulating the tumor necrosis factor Tnfrsf14 and the inflammation‐related gene Hspb7. This may be due to BPM generating substantial H_2_, which can cause extensive DNA damage by inhibiting mitochondrial function and blocking adenosine ATP synthesis, thereby promoting cell apoptosis. Additionally, immunosuppressive chemokines Cxcl12 and Cxcl13 were downregulated, while the immunostimulatory chemokine Ccl11 was upregulated. T‐cell activation‐inducing factors Cd8a, Icos, Gzmb, and Zap70 were also upregulated, suggesting that BPM by US excitation may activate the immune system and induce a strong immune response in the TME.

Gene ontology (GO) enrichment analysis revealed the biological processes involved after synergistic treatment. As shown in Figure 6d, dysregulated genes significantly participated in various biological processes, including immune response, T‐cell migration, lymphocyte migration, receptor surface signaling pathways, cell proliferation, and necrosis. Furthermore, we conducted a Kyoto Encyclopedia of Genes and Genomes (KEGG) pathway enrichment analysis to understand how BPM exerts its effects through H_2_ and O_2_ in tumor cells. KEGG analysis showed that the DEGs were significantly enriched in 10 cellular pathways (Figure 6e). Among them, the enrichment of MAPK signaling, T‐cell receptor signaling pathway, transcriptional misregulation in cancer, cGMP‐PKG signaling pathway, and PI3K‐Akt signaling pathway indicated that the synergistic treatment could effectively inhibit tumor proliferation and metastasis while activating the immune system to elicit a robust immune response. A chord diagram (Figure 6f) and a Sankey diagram (Figure 6g) revealed the correlation between DEGs and GO pathways and KEGG pathways, respectively.

## 3 Conclusion

In summary, this study successfully designed and validated a sonocatalyst, BPM, for in situ H_2_ and O_2_ generation in the TME. Under ultrasonic stimulation, BPM demonstrated charge separation and electron transfer efficiency, improving water‐splitting efficiency and ensuring a continuous supply of H_2_ and O_2_. The production of H_2_ effectively disrupted the mitochondrial function of tumor cells, leading to energy metabolism exhaustion and apoptosis, while the generation of O_2_ alleviated hypoxia within the tumor and enhanced the tumor's sensitivity to treatment. The experimental results showed that BPM not only inhibited tumor cell proliferation through direct catalytic action but also further impaired the tumor's antioxidant defenses by consuming GSH. Additionally, BPM significantly enhanced immune responses within the TME, promoting DCs maturation, T‐cell activation, and macrophage polarization, thereby reversing the immunosuppressive state and triggering a strong antitumor immune response. Moreover, BPM exhibited good biocompatibility and safety in both in vitro and in vivo experiments. In clinic, most solid tumors are hypoxic. In situ catalytic water splitting can relieve tumor hypoxia and provide auxiliary treatment. Eliminating hypoxia can enhance the sensitivity of tumors to other treatments and improve the efficacy. These findings support the design rationale of BPM as a gas‐immunotherapy combination strategy, offering an approach for future cancer treatments.

## 4 Experimental Section

### Synthesis of POM‐Mo_2_C (PM)

Disperse 1 mg of Mo_2_C in 15 mL of distilled water and stir rapidly at 1000 rpm. Then, quickly add 1 mL of 30% H_2_O_2_ and allow the reaction to proceed for 12 h. After completion, centrifuge the mixture and retain the supernatant. Repeat the centrifugation process 4 to 5 times. Finally, freeze‐dry the collected supernatant to obtain POM‐Mo_2_C (blue powder), which was then stored in a dry environment.

### Synthesis of BiF_3_‐POM‐Mo_2_C (BPM)

Dissolve 0.2 mmol of Bi(NO_3_)_3_·5H_2_O and 40 mg of POM in 2 mL of PEG400 to prepare a blue mixed solution. Then, rapidly add 5 mL of EG solution containing 0.4 mmol of NH_4_F and stir vigorously at 25 °C for 120 s. The reaction was terminated by adding an equal volume of deionized water. The resulting mixture was centrifuged and washed with deionized water three times. Finally, the obtained BPM was dispersed in deionized water and stored at 4 °C.

### Cell Culture and Grouping

4T1 murine breast cancer cells were obtained from the Shanghai Institute of Biological Sciences, Chinese Academy of Sciences, and cultured in RPMI‐1640 medium supplemented with 10% fetal bovine serum. The cells were maintained at 37 °C in a humidified incubator with 5% CO_2_ (Thermo BB150, USA). The cells were divided into six experimental groups: 1) Control group (untreated cells); 2) US group (cells treated with ultrasound only); 3) BiF_3_+US group (cells treated with BiF_3_ and ultrasound); 4) PM+US group (cells treated with PM and ultrasound); 5) BPM group (cells treated with BPM only); and 6) BPM+US group (cells treated with BPM and ultrasound). The US conditions were 0.7 W cm^−2^, 1 MHz frequency, 60% duty cycle, and 5 min of exposure.

### Establishment of Tumor Model

To establish a murine breast cancer model, 5‐week‐old female BALB/c mice were injected subcutaneously with 4T1 cells (2 × 10^6^ cells mL^−1^, 100 µL) into the forelimb. The animal facility was accredited by the Shanghai Municipal Commission of Science and Technology, China (SYXK 2020‐0006). The experiments followed the guidelines of the Animal Ethics Committee of Shanghai East Hospital, China (Protocol No. 2023‐036‐01).

### Statistical Analysis

Statistical analysis was performed using IBM SPSS Statistics 25 software. A t‐test was used to assess differences between groups, and statistical significance was indicated as follows: *p < 0.05, **p < 0.01, ***p < 0.001. The data from parallel experiments were expressed as mean ± standard deviation (mean ± SD).

## Conflict of Interest

The authors declare no conflict of interest.

## Supporting information



Supporting Information

## Data Availability

The data that support the findings of this study are available from the corresponding author upon reasonable request.
