# Supplementary material for: Ternary Schottky Junction for Sonocatalytic Water Splitting in Gas‐Immunotherapy‐Mediated Cancer Treatment
Source: Adv Sci (Weinh). 2025 Feb 8;12(13):2413519. doi: 10.1002/advs.202413519 (PMC11967844; doi:10.1002/advs.202413519)
Supplement: Supplementary file 1 — Supporting Information [file ADVS-12-2413519-s001.docx]

Supporting Information

**Ternary** **Schottky Junction for Sonocatalytic Water Splitting in Gas-Immunotherapy-Mediated Cancer Treatment**

*Rui Zhang^†^, Qian Wang^†^, Junjie Pan, Jun Du, Han Yang, Bingfeng Wang, Yuhao Li*, Yuqing Miao*, Xumin Hou*, Jingxiang Wu*, Qing Miao**

R. Zhang, X Hou, J. Wu, Q. Miao

Department of Anesthesiology, Shanghai Chest Hospital, School of Medicine, Shanghai Jiao Tong University, Shanghai 200030, China.

R. Zhang, Q. Wang, J. Pan, J. Du, H. Yang, Y. Li, Y. Miao

Institute of Bismuth Science, School of Materials and Chemistry, Shanghai Collaborative Innovation Center of Energy Therapy for Tumors, University of Shanghai for Science and Technology, Shanghai 200093, China.

B. Wang

College of Materials and Energy, South China Agricultural University, Guangzhou 510631, China.

† Contributed equally

Corresponding Authors:

Yuhao Li: E-mail yhli@usst.edu.cn

Yuqing Miao: E-mail yqmiao@usst.edu.cn

Xumin Hou: E-mail hxmchest@163.com

Jingxiang Wu: E-mail wu_jingxiang@sjtu.edu.cn

Qing Miao: E-mail miaoqmz@163.com

**Supplementary Experimental Section**

*1. Materials*

Bi(NO_3_)_3_·5H_2_O (99.9%), NH_4_F (99.99%), Mo_2_C (99.95%), and methylene blue (MB) were obtained from Adamas (China). IR-780 iodide was sourced from Sigma-Aldrich (USA). Ethylene glycol (EG) (≥99.8%), Na_2_SO_4_ (≥99%), [Fe(CN)_6_]^3−/4−^, KCl, H_2_O_2_ (30%), and dimethyl sulfoxide (DMSO, ≥99.0%) were purchased from General Reagent (China). Reagents for biological assays, including cell counting kit-8 (CCK-8), JC-1 mitochondrial membrane potential assay kit, DCFH-DA, 4% paraformaldehyde, annexin V-FITC/PI apoptosis detection kit, DAPI staining solution, anti-rabbit 488, and ATP assay kit, were procured from Beyotime Biotechnology (China). Triton X-100, anti-HIF-1α antibody, anti-CRT antibody, and anti-HMGB1 antibody were supplied by Servicebio (China). Additional cell culture reagents, such as trypsin-EDTA (0.25%), PBS, red blood cell lysis buffer, penicillin-streptomycin, and RPMI-1640, were acquired from Adamas (China). The anti-mouse CD16/CD32 (Fc shield) antibody was purchased from Tonbo Biosciences (USA), while various flow cytometry antibodies, including Zombie Violet Fixable Viability Kit, PE anti-mouse CD4, FITC anti-mouse CD3, FITC anti-mouse CD11b, APC anti-mouse CD8, PE anti-mouse CD11c, PE anti-mouse CD206, APC-Cy7 anti-mouse CD45, Cy7 anti-mouse F4/80, APC anti-mouse CD86, and PerCP-Cy5.5 anti-mouse CD80, were obtained from BioLegend (China).

*4. Synthesis of BiF_3_-Mo_2_C*

Disperse Mo_2_C in deionized water and use an ultrasonic cell crusher (Scientz 650E, China) to break the particles. Centrifuge the dispersed solution at 7,000 rpm, separate the supernatant, and freeze-drying to obtain the product. Dissolve 0.2 mmol of Bi(NO_3_)_3_·5H_2_O and 20 mg of broken Mo_2_C in 2 mL of PEG400 to prepare a black mixed solution. Then, quickly add 5 mL of EG solution containing 0.4 mmol of NH_4_F and stir vigorously at 25 ℃ for 120 s. Terminate the reaction by adding an equal volume of deionized water. Centrifuge the mixture at 10,000 rpm for 10 minutes (min) and wash it with deionized water three times. Finally, disperse the obtained BiF_3_-Mo_2_C in deionized water and store it at 4 ℃.

*5. Characterization*

Structural analysis was performed using transmission electron microscopy (FEI Tecnai G2 F20, USA; Hitachi HT 7800, Japan). The elemental chemical states were characterized via X-ray photoelectron spectroscopy (Thermo K-Alpha, USA). Hydrodynamic particle size was determined with a nanoparticle size analyzer (Malvern Zetasizer Lab, UK). Fourier-transform infrared (FT-IR) spectra were acquired using an infrared spectrometer (IRTracer-100, Japan), while absorption properties were assessed using a UV–visible spectrophotometer (Shimadzu UV-1900, Japan). The specific surface area and pore size distribution were measured using a specific surface area analyzer based on the Brunauer−Emmett−Teller (BET) method (Autosorb iQ, Quantachrome, USA).

*6. Detection of H_2_ generation in solution*

*6.1 Measurement of H_2_ volume standard curve*

Using a micropipette, inject different volumes of H_2_ (10, 20, 30, 40, 50 µL) into the H_2_ channel of the gas chromatograph (GC-7920, CEAULIGHT, China). Record the signal values for different volumes of H_2_ within 0.5 to 0.8 min and fit the peak values to obtain the H_2_ volume standard curve.

*6.2 H_2_ detection*

Disperse BPM (1 mg mL^−1^) in deionized water, then inject 10 mL of the dispersion into a 20 mL penicillin bottle and seal it. Purge the liquid with nitrogen for 30 min to remove other gases from the liquid and the bottle, and then reseal it. The sample is exposed to ultrasonic excitation at various power levels (1 MHz, 60% duty cycle, WED-100, China), and the signal values within 0.5 – 0.8 min are recorded using a gas chromatograph. The peak values are used to calculate the amount of H_2_ generated under different ultrasonic powers and durations by referencing the H_2_ volume standard curve.

*7. Detection of O_2_ generation in solution*

Disperse BPM (1 mg mL^−1^) in deionized water, and inject 10 mL of the dispersion into a 20 mL penicillin bottle, sealing it tightly. Purge the liquid with nitrogen for 30 min to remove any gases present in the liquid and bottle, then reseal the bottle. The sample is exposed to ultrasonic excitation at different power levels (1 MHz, 60% duty cycle), and an oxygen meter (ST300D, OHAUS, USA) is used to record the O_2_ concentration at various time points.

*8. Electrochemical performance testing*

All electrochemical measurements were conducted in a standard three-electrode system using a CHI660E electrochemical workstation (CHI-Instrument, China) in a 1 M Na_2_SO_4_ solution (pH = 7). A platinum sheet, Ag/AgCl electrode, and fluorine-doped tin oxide (FTO) glass substrate electrode (1 × 1 cm) served as the counter electrode, reference electrode, and working electrode, respectively.

*9. Photocurrent and sonocurrent detection*

The photocurrent of BPM, BiF_3_-Mo_2_C, and BiF_3_ was measured in PBS solution (pH 7.4, 0.01 M) using an electrochemical workstation. The system consisted of a working electrode (containing 200 mL of BPM, PM, and BiF_3_ (1 mg mL^−1^) on conductive glass), a reference electrode, and a counter electrode (platinum sheet). The photocurrent was recorded with or without continuous illumination from a 300 W xenon lamp (100 mW cm^−2^). Under the same procedure, a 300 W xenon lamp was replaced to ultrasound (1 W cm^−2^) for collecting sonocurrent of BMP.

*10. Mott–Schottky curve detection*

Mott–Schottky curves for BPM, Mo_2_C, and BiF_3_ were measured using an electrochemical workstation in a 0.5 M Na_2_SO_4_ solution. Working electrodes were prepared by depositing BPM, PM, and BiF_3_ (100 µg mL^−1^, 200 µL) onto the conductive glass. The Mott–Schottky curves were obtained at a frequency of 1000 Hz, within a voltage range of −2 to 1 V.

*11. Electrochemical impedance detection*

The electrochemical impedance of BPM, PM, and BiF_3_ was measured in an electrolyte solution containing 1 mM K_3_[Fe(CN)_6_], 1 mM K_4_[Fe(CN)_6_], and 0.5 M KCl. Working electrodes were prepared by depositing BPM, BiF_3_-Mo_2_C, and BiF_3_ (1.0 mg mL^−1^, 10 µL) onto glassy carbon electrodes, and the resulting alternating current impedance spectra were recorded.

*12. Calculation*

The structure of BiF_3_ is derived from CCDC (ICSD: 655136), and the structure of Mo_2_C is derived from the Materials project (MP-571589). Firstly, geometry optimization was performed on the above structure using the PBE function of GGA method (Energy cutoff 500 eV, SCF tolerance fine, k-point 2 × 2 × 1). After optimization, calculate the energy band and density of states. The entire calculation process is completed by the CASTEP module of Material Studio 8.0.

*13. Contact angle measurement*

BPM (200 mL, 1 mg mL^−1^) was loaded onto a piece of FTO glass, use a contact angle measuring instrument (OCA25, Dataphysics, Germany) to record real-time images of deionized water contacting the BPM surface, and analyze the contact angle through software. The contact of pure water on the surface of FTO is used as the control group.

*14. Polarization curve and Tafel slope measurement*

Disperse 5 mg of BPM, Mo_2_C, and BiF_3_ in 5 mL of deionized water and sonicate for 30 min to obtain a uniform suspension. Pipette 100 µL of each dispersion onto the surface of FTO glass substrate electrodes, dry at room temperature, and then proceed with electrochemical testing. Polarization curves were obtained using linear sweep voltammetry (LSV) under ultrasonic power conditions of 1.5 W cm^−2^ and 1.0 MHz, with a scan rate of 10 mV s^−1^. Tafel slope plots were derived from the corresponding LSV data. The results were referenced to a reversible hydrogen electrode (RHE) using the following formula: E_(V vs RHE)_ = E_(V vs Ag/AgCl)_ + 0.197 V + 0.059 × pH.

*15. GSH content evaluation*

A 5,5’-dithiobis-(2-nitrobenzoic acid) (DTNB) probe was used to monitor GSH levels in different experimental groups. In each group, 10 mM GSH was dissolved in PBS (pH 7.4). The control group received no treatment, while the US group was treated with ultrasound for 10 min. In the BiF_3_, BPM, and BPM+US groups, materials were used at a concentration of 200 µg mL^−1^, and ultrasound treatment (0.7 W cm^−2^, 10 min) was applied in the BPM+US group. After treatment, the samples were incubated for 3 h at 37 ℃, then centrifuged for 10 min at 13,500 rpm to collect the supernatant. The relative GSH content in the supernatant was measured using a total glutathione assay kit (Beyotime, China) according to the manufacturer’s instructions.

*16. Degradation behavior of BOSC*

The degradation behavior of BPM was studied in pH 5.5/H_2_O_2_/GSH (BPM: 100 µg mL^−1^, GSH: 10 mM, H_2_O_2_: 5 mM) physiological buffer systems. After 0 to 24 h, the morphology of BPM was also observed by a transmission electron microscope.

*18. Cytotoxicity assay and in vitro therapeutic effects*

In vitro cytotoxicity tests were performed on each group of cells, and cell viability was assessed using the Cell Counting Kit-8 (CCK-8). 293T and 4T1 cells were seeded in 96-well plates at a density of 5,000 cells per well. After adding BiF_3_, PM, or BPM (100 μg mL^−1^) dispersed in RPMI-1640 medium, the cells were co-incubated for 24 or 48 h before being subjected to US treatment. Following US exposure, the cells were incubated for an additional 24 h at 37 ℃, and cell viability was measured using the CCK-8 assay. According to the kit’s instructions, the cells were washed with PBS to remove excess materials, and then 100 μL of RPMI-1640 containing 10% CCK-8 reagent was added to each well. After 2 h of incubation at 37 ℃, absorbance at 450 nm was measured using a microplate reader to determine cell viability.

*19. Intracellular ROS detection*

The production of reactive oxygen species (ROS) in the cells was monitored using the 2’,7’-dichlorodihydrofluorescein diacetate (DCFH-DA) probe. 4T1 cells were seeded into 12-well plates, and after the specified treatments, the cells were incubated with DCFH-DA in the dark for 30 min as per the manufacturer’s instructions. The cells were then washed with PBS, and the green fluorescence of DCFH-DA was observed and recorded using a fluorescence microscope (IX73, Olympus, Japan).

*20. CRT exposure and HMGB1 release detection*

Immunofluorescence was used to detect CRT exposure and HMGB1 release. 4T1 cells (5×10^3^ cells) were seeded into 24-well plates and cultured for 12 h. After the various treatments were applied for 6 h, the cells were fixed with 4% paraformaldehyde for 30 min and washed with PBS. The cells were then treated with 0.3% Triton X-100 for 20 min and blocked with 3% BSA for 2 h. Subsequently, the cells were incubated overnight at 4 ℃ with an anti-CRT antibody (1:500) or anti-HMGB1 antibody (1:1000). The next day, the cells were incubated with anti-rabbit 488 secondary antibody for 2 h and stained with 4’,6-diamidino-2-phenylindole (DAPI) for 15 min. CRT exposure and HMGB1 release were observed using a fluorescence microscope.

*21. ATP release detection*

ATP detection reagents were prepared according to the instructions provided with the ATP assay kit. 4T1 cell suspensions (1×10^5^ cells) were seeded into 6-well plates and cultured for 24 h. After treatment in each group for 24 h, 100 μL of ATP assay working solution and 20 μL of culture supernatant were added to 96-well plates (n = 5). ATP release was quantified using a microplate reader according to the manufacturer’s instructions.

*22. Mitochondrial membrane potential measurement*

The JC-1 staining solution and wash buffer were prepared according to the instructions provided with the JC-1 Mitochondrial Membrane Potential Assay Kit. 4T1 cells were seeded into 24-well plates and cultured for 12 h, followed by the corresponding treatments. After 24 h, 0.5 mL of JC-1 staining solution was mixed with 0.5 mL of culture medium, and the cells were incubated at 37 ℃ for 20 min. The cells were then washed with wash buffer, and changes in mitochondrial membrane potential were observed and captured using a fluorescence microscope.

*23. In vitro γ-H2AX detection*

4T1 cells were seeded into 24-well plates and cultured for 12 h before treatment according to the experimental protocols for each group. Staining was performed according to the instructions provided in the γ-H2AX assay kit. After 24 h of treatment, cells were washed once with PBS and then fixed for 15 min with a fixative solution. Next, an immunostaining blocking solution was added, and the cells were incubated at room temperature for 15 min. After removing the blocking solution, the cells were incubated overnight at 4 ℃ with γ-H2AX rabbit monoclonal antibody. The following day, the cells were incubated with anti-rabbit 488 secondary antibody for 1 h at room temperature and counterstained with DAPI to label the nuclei. Images were captured using a fluorescent microscope.

*24. Intracellular H_2_ release measurement*

4T1 cells were seeded into 24-well plates and incubated for 12 h with the designated materials. After incubation, the cells were further incubated with 40 µM methylene blue (MB) solution for 30 min in a cell incubator. After incubation, the cells were gently washed three times with PBS. For the groups requiring US treatment, US was applied for 5 min, after which cell images were captured under a microscope.

*26. In vivo fluorescence imaging*

IR780 was loaded into BPM for in vivo fluorescence imaging. IR780 (6 mg) and BPM (1 mg mL^−1^ methanol solution, 3 mL) were thoroughly mixed and stirred in the dark for 24 h. The BPM-IR780 complex was collected by centrifugation (10,000 rpm, 10 min) and washed with PBS. Before use, BPM-IR780 was dispersed in PBS. Mice were injected with BPM-IR780 (2 mg mL^−1^, 100 μL) via the tail vein, and fluorescence images were captured at various time points (1, 3, 6, 12, and 24 h) using a small animal imaging system (PerkinElmer IVIS LuminaⅢ, USA) (λ_ex_ = 640 nm, λ_em_ = 800 nm). After imaging, the mice were euthanized, and major organs and tumor tissues were collected. The fluorescence of the organs and tumors was observed, and fluorescence intensity was quantified using Image J software.

*27. Hemolysis assay*

Mouse eye blood (1 mL) was collected and mixed with PBS (2 mL). After standing for 1 h, the mixture was centrifuged at 1,000 rpm for 5 min. The red blood cells were washed with PBS until the supernatant was clear. The red blood cells were then resuspended in 4 mL of PBS. A 0.15 mL aliquot of the red blood cell suspension was mixed with 1 mL of deionized water (positive control), PBS (negative control), or BPM solutions at varying concentrations (25 – 200 µg mL^−1^). After centrifugation, the absorbance at 570 nm was measured to calculate the hemolysis rate.

*28. Hematological analysis*

BALB/c mice were intravenously injected with BPM (2 mg mL^−1^, 100 μL), and blood samples were collected on day 14 for hematological analysis. The mice were then euthanized, and their hearts, livers, spleens, lungs, and kidneys were dissected and fixed in formalin. Tissue sections were prepared and stained with hematoxylin and eosin (H&E) for histological examination.

*29. In vivo synergistic tumor therapy*

Once the tumor diameter reached 5 mm, the mice were randomly divided into six treatment groups (n = 5): (1) Control group, (2) US group, (3) BiF_3_+US group, (4) PM+US group, (5) BPM group, and (6) BPM+US group. Groups (3), (4), (5), and (6) were injected intravenously with BiF_3_, PM, and BPM solutions (2 mg mL^−1^, 0.2 mL). For groups (2), (3), (4), and (6), ultrasound irradiation (0.7 W cm^−2^, 1 MHz, 60% duty cycle, 5 min) was applied 12 h after injection. After 24 h, tumor tissues from groups (1) and (6) were collected for RNA sequencing. To evaluate the therapeutic efficacy of the different treatment groups, the mice’s body weight and tumor volume were measured every two days. Finally, tissue sections from the tumors and major organs were prepared for histological and histochemical analysis.

*30. Flow cytometry analysis of tumor immune microenvironment*

The tumor, spleen, lymph nodes, and bone marrow from each experimental group of mice were dissected and analyzed by flow cytometry. In brief, the tumor tissues were processed using an immune cell tumor dissociation kit, while spleen and bone marrow tissues were treated with red blood cell lysis buffer. Anti-CD16/CD32 antibodies were dissolved in PBS and used to block Fc receptors on immune cells. The immune cells were then stained for 30 min with antibodies against CD45, CD3, CD4, CD8, CD11c, CD11b, F480, CD206, CD80, and CD86. After washing with PBS, the cells were stained with the zombie violet viability kit, followed by fixation. The cells were then analyzed by flow cytometry (BD Melody, USA), and the data were processed using FlowJo software.


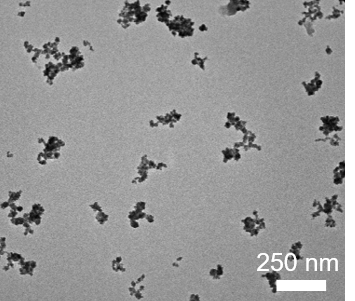


**Figure S1.** TEM image of BPM.


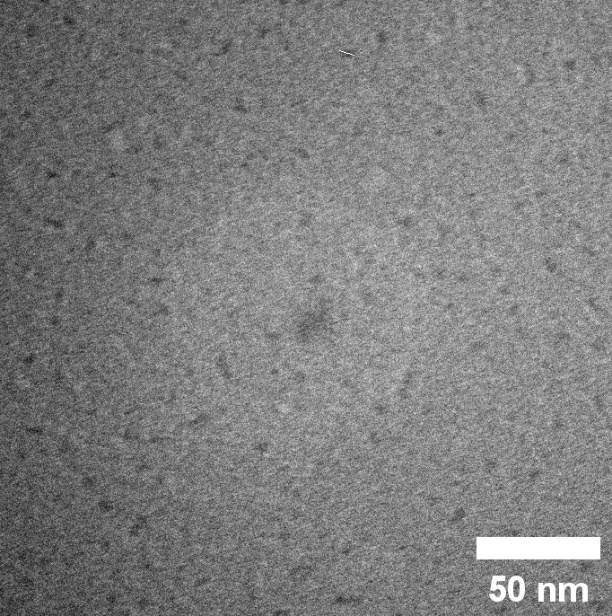


**Figure S2.** TEM image of POM-Mo_2_C.


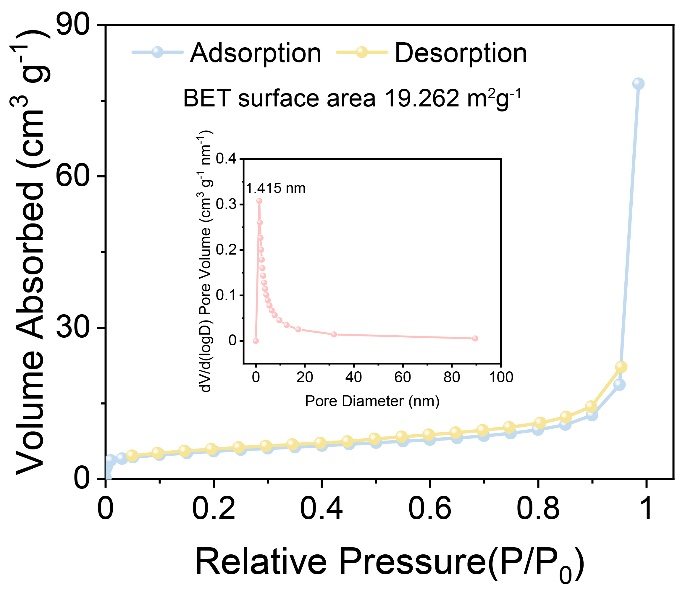


**Figure S3.** BET result of BPM (inset: average pore size of BPM).


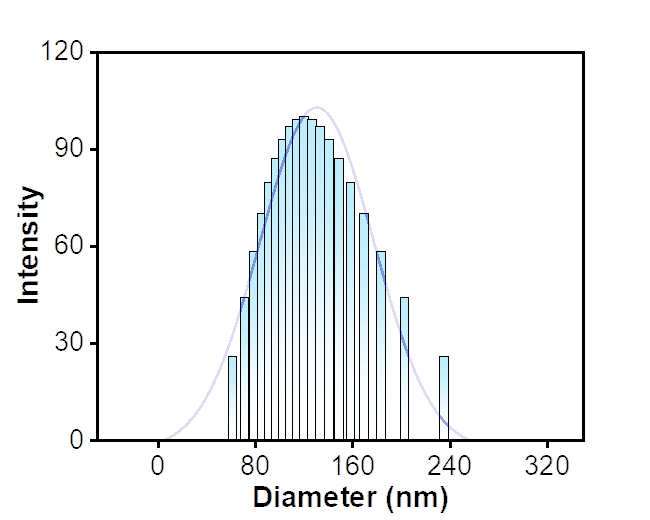


**Figure S4.** DLS analysis of BPM in PBS.


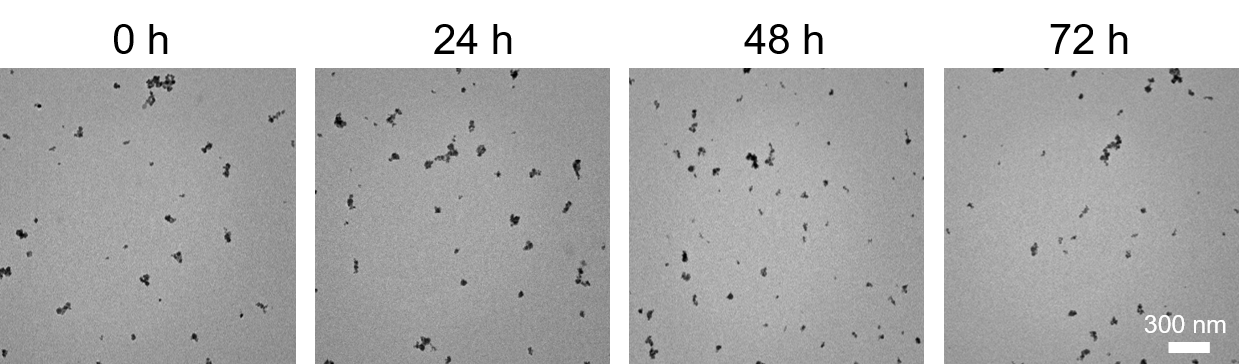


**Figure S5.** Time-dependent TEM image of BPM. (BPM is stored in pH 7.4 buffer solution)


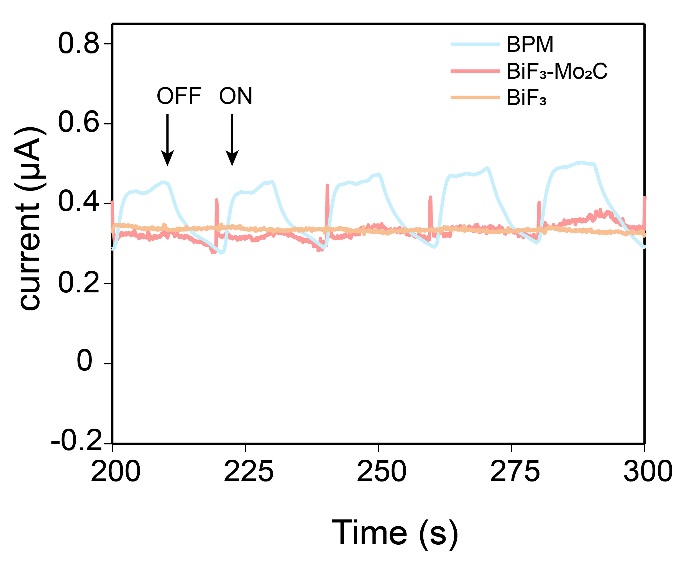


**Figure S6.** Time-dependent sonocurrent response of BiF_3_, BiF_3_-Mo_2_C, and BPM.


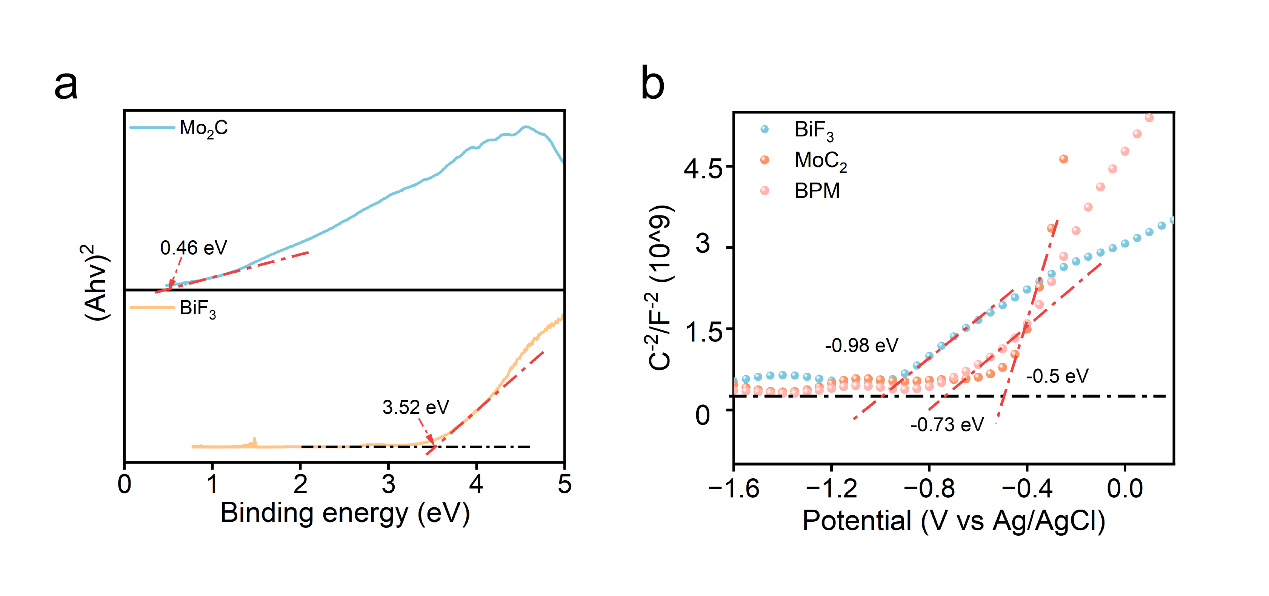


**Figure S7.** (a) Band gap of BiF_3_ and Mo_2_C; (b) Mott–Schottky curves of BiF_3_, Mo_2_C, and BPM.


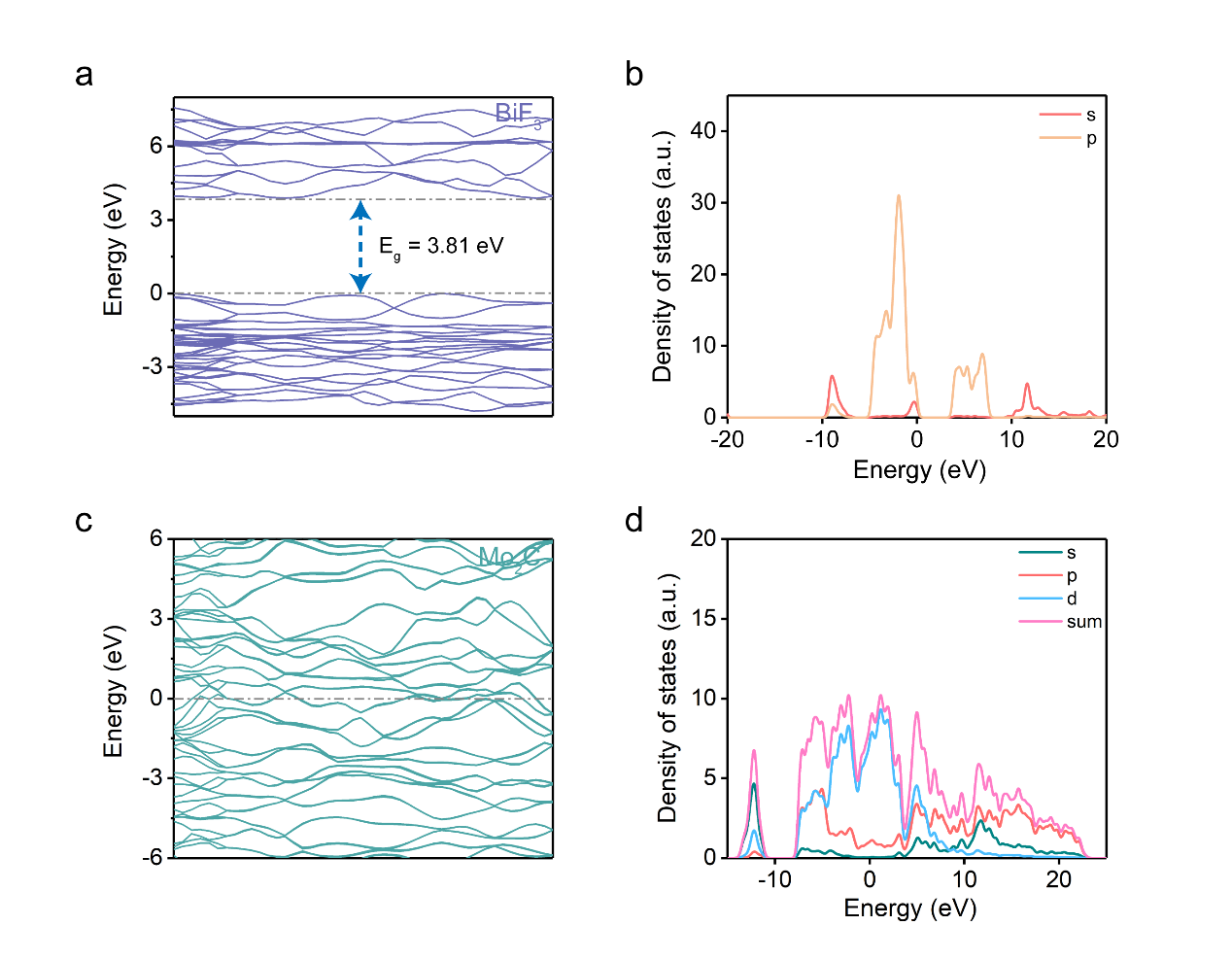


**Figure S8.** (a) Calculated band diagram of BiF_3_; (b) projection electron density of states (PDOS) of BiF_3_; (c) the calculated band diagram of Mo_2_C; (d) PDOS of Mo_2_C.


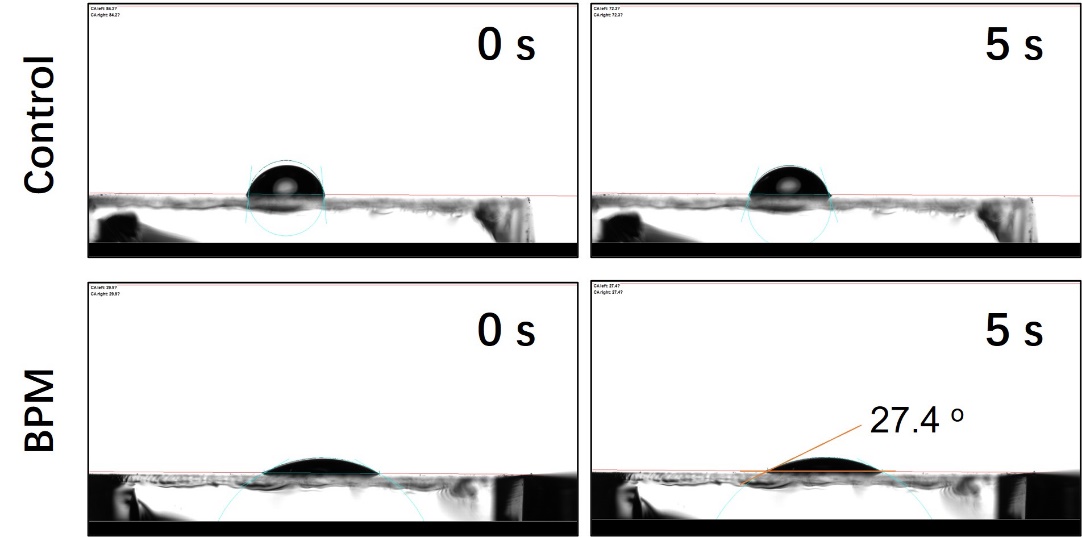


**Figure S9.** Contact angle of deionized water on FTO glass surface (Control) and BPM coated on FTO surface at 0 s and 5 s.


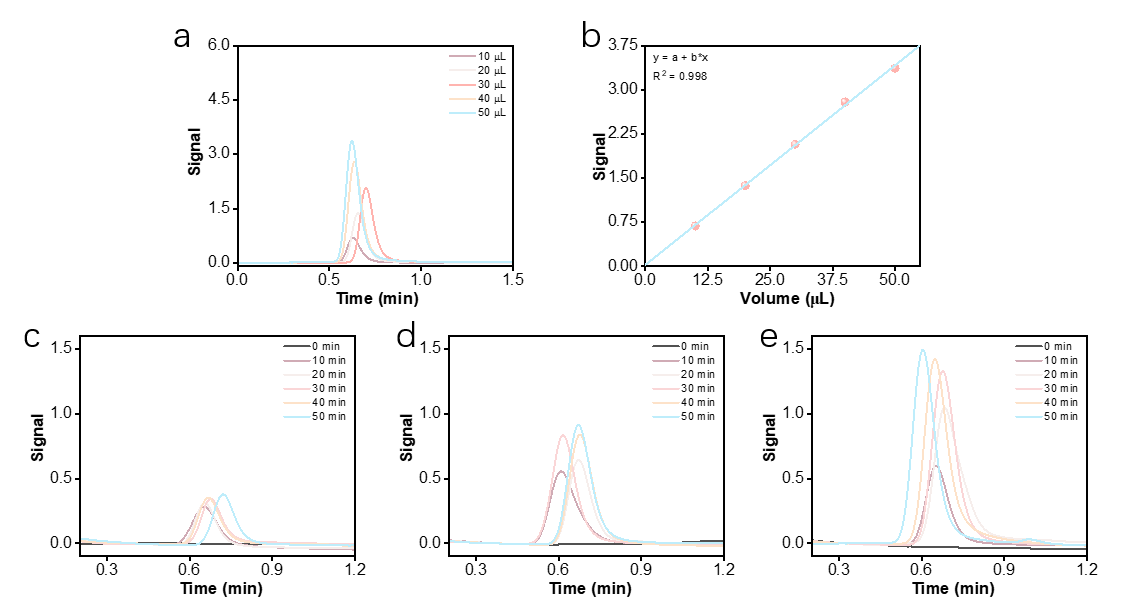


**Figure S10.** (a) Gas chromatograms (GC) of H_2_ with different volumes. (b) The standard curve was obtained from the volume of H_2_ and its signal intensity in the GC. GC spectra of H_2_ generated by BPM at different ultrasound power densities (c) 1 W cm^−2^, (d) 1.5 W cm^−2^, (e) 2 W cm^−2^ over times.


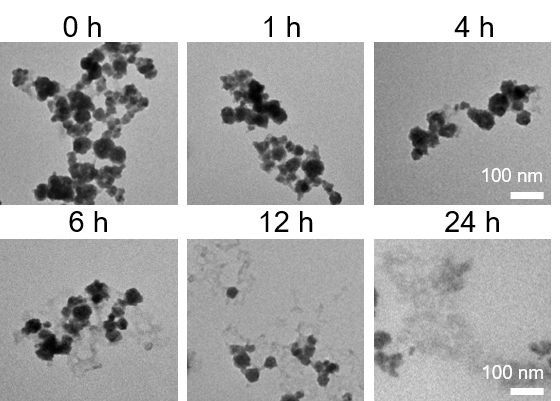


**Figure S11.** TEM images of BPM incubated with GSH (10 mM) in pH 5.5/H_2_O_2_ (10 mM) physiological buffer system over time.


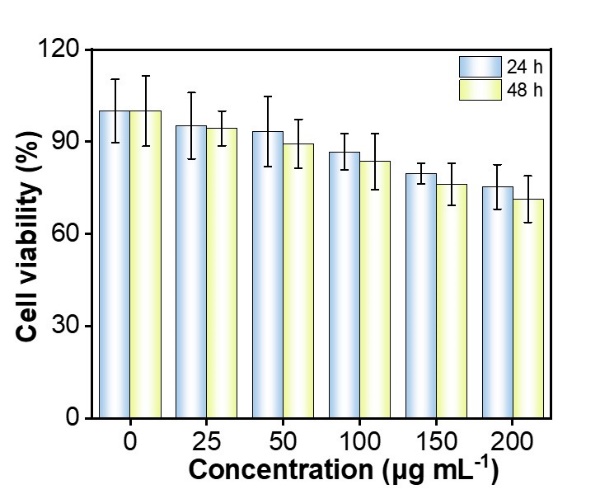


**Figure S12.** Cell viability of 293T cells after co-incubation with BPM for 24 and 48 h, respectively.


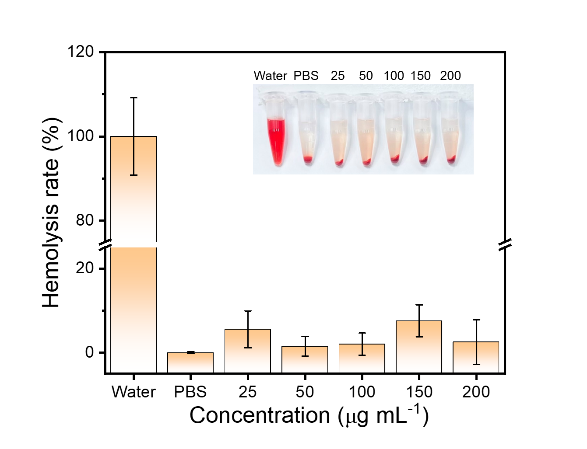


**Figure S13.** Hemolysis analysis results of different concentrations of BPM (inset: hemolysis images of red blood cells in corresponding groups).


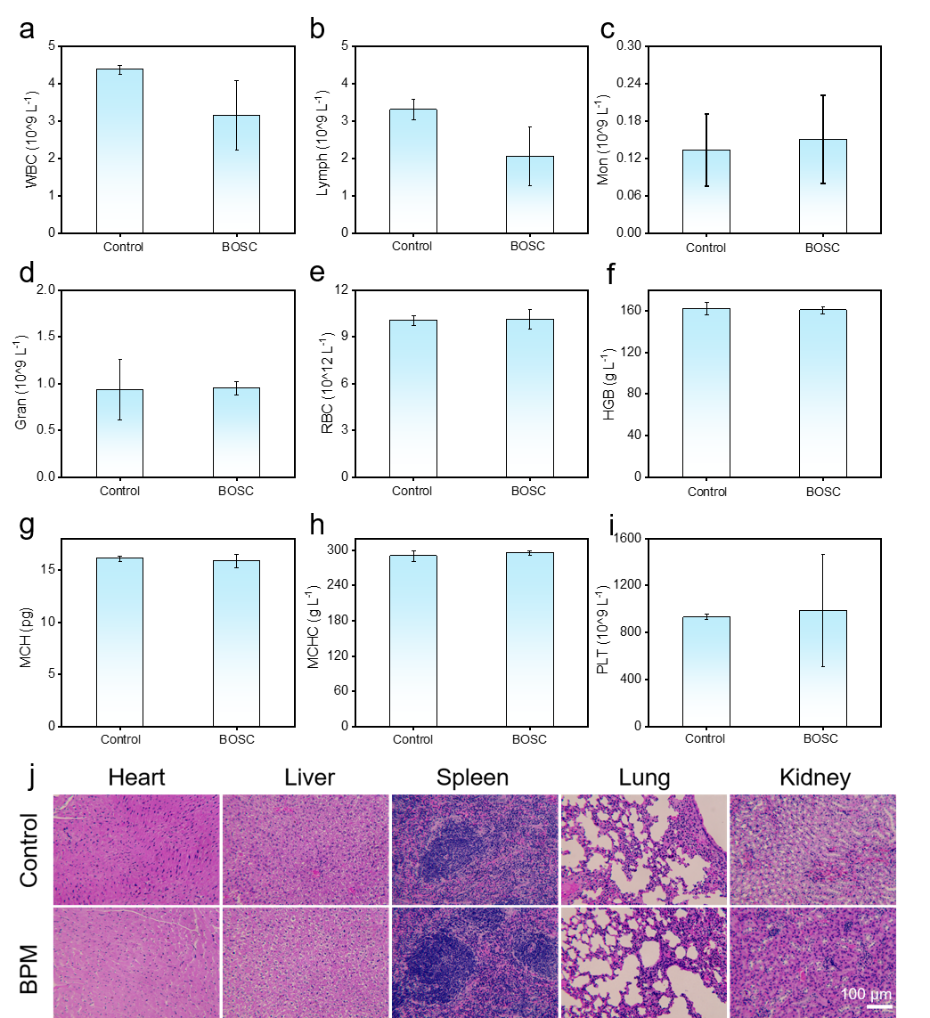


**Figure S14.** Complete blood count data of BALB/c female mice treated with BPM (2 mg mL^−1^, 100 µL) and PBS (control group, 100 µL) intravenous injection for 21 days. (a) White blood cell (WBC), (b) lymphocyte percentage (Lymph), (c) monocyte percentage (Mon), (d) neutrophil percentage (Gran), (e) red blood cell count (RBC), (f) hemoglobin (HGB), (g) mean corpuscular hemoglobin (MCH), (h) mean corpuscular hemoglobin concentration (MCHC), (i) platelet count (PLT), (j) H&E stained sections of major organs in mice after BPM and PBS (control group) injection, including heart, liver, spleen, lung, and kidney.


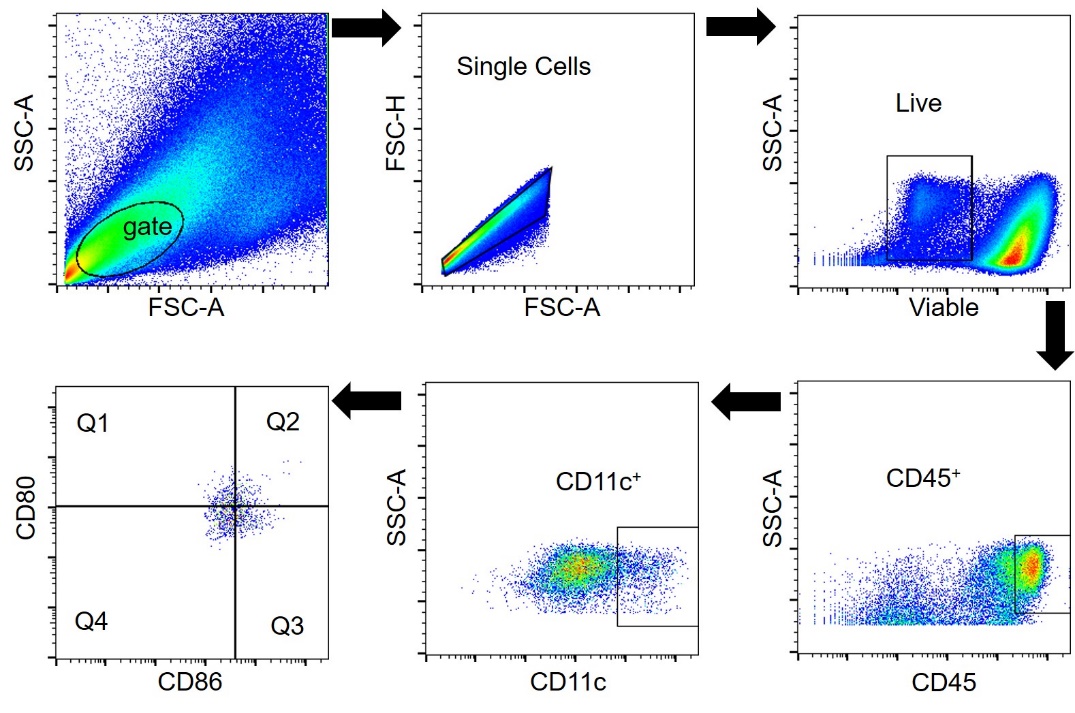


**Figure S15.** Gating strategy of fluorescence-activated cell sorting analysis for measuring mature DCs in the CD11c^+^ cells.


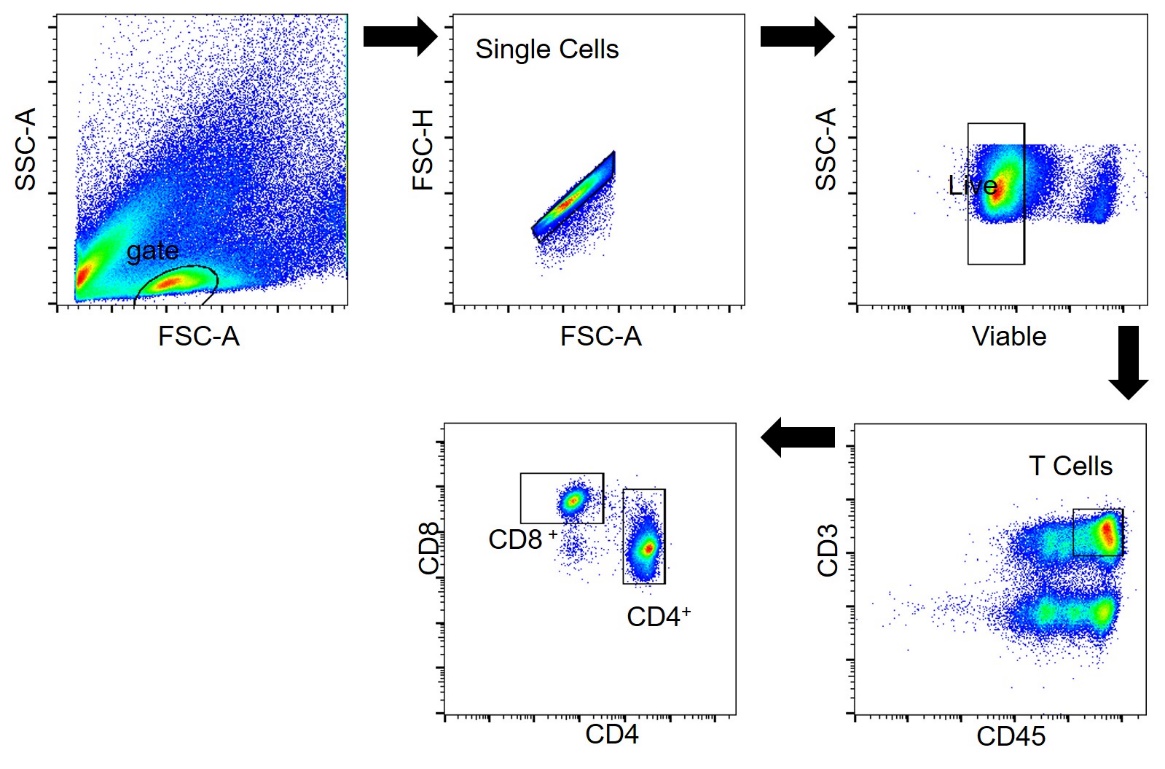


**Figure S16.** Gating strategy for fluorescence-activated cell sorting analysis to measure CD8^+^ and CD4^+^ cells within the CD3^+^ cell population.


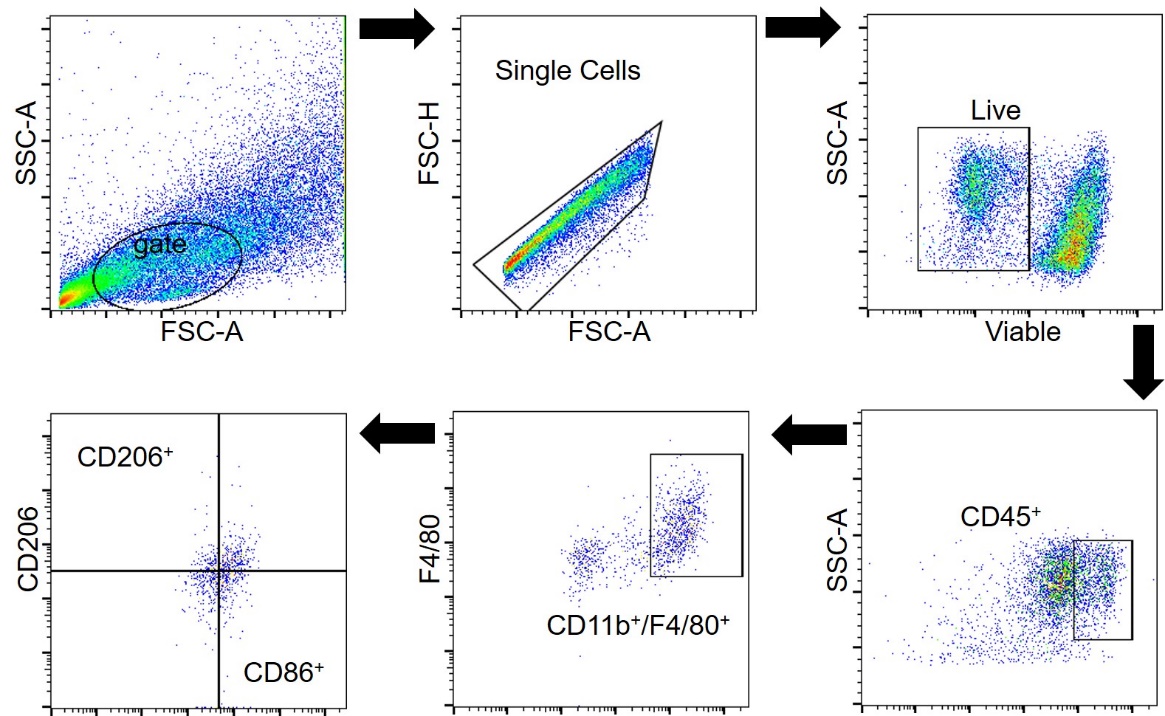


**Figure S17.** Gating strategy for fluorescence-activated cell sorting analysis to measure CD86 and CD206 cells within the CD11b/F4/80^+^ cell population.
